# Comparison of concurrent, resistance, or aerobic training on body fat loss: a systematic review and meta-analysis

**DOI:** 10.1080/15502783.2025.2507949

**Published:** 2025-05-22

**Authors:** Kworweinski Lafontant, Alexa Rukstela, Ardis Hanson, Janet Chan, Yasamian Alsayed, Wayne A. Ayers-Creech, Cassidy Bale, Yuto Ohigashi, John Solis, Gretchen Shelton, Indira Alur, Cassandra Resler, Andrew Heath, Savannah Ericksen, Scott C. Forbes, Bill I. Campbell

**Affiliations:** a University of South Florida, Performance & Physique Enhancement Lab, Exercise Science Program, Tampa, FL, USA; b University of Central Florida, Physiology of Work and Exercise Response (POWER) Lab, Institute of Exercise Physiology and Rehabilitation Sciences, Orlando, FL, USA; c University of South Florida, USF Health Libraries, Tampa, FL, USA; d Brandon University, Department of Physical Education Studies, Brandon, Manitoba, Canada

**Keywords:** Interference effect, body composition, lean body mass, bodyfat, exercise selection

## Abstract

**Background:**

This systematic review and meta-analysis compared the differential effects of resistance training (RT), aerobic training (AT), and concurrent training (CT) on body mass and body fat loss in metabolically healthy individuals.

**Methods:**

A systematic search of PubMed, SportDiscus, and Web of Science databases for randomized controlled trials published between January 1980 and January 2023, comparing RT, AT, and CT in healthy adults was conducted. Primary outcomes of interest included changes in fat mass and body fat percentage; secondary outcomes were body mass and fat-free mass (FFM). Sub-analyses on intervention duration (< or ≥ 10 weeks), CT timing (aerobic and resistance exercises done on the same day versus different days within a week), and workload matching (equating workloads between AT, RT, and CT), were conducted. Study protocols followed PRISMA 2020 guidelines and were pre-registered on PROSPERO (CRD42023396530).

**Results:**

In total, 36 studies with 1564 participants were included in the systematic review, with only 31 studies included in the meta-analysis due to missing data. For studies lasting at least 10 weeks, AT outperformed RT in reducing body mass (mean difference (MD) = -1.82 kg [95% CI = -2.72 to −0.93]; *p* < 0.001) and fat mass (MD = -1.06 kg [95% CI = -1.88 to −0.24]; *p* = 0.01) but led to less FFM retention (MD = - 0.88 kg [95% CI = -1.73 to −0.03], *p* = 0.04). CT reduced significantly more fat mass compared to RT (MD: −1.09 kg [95% CI = -0.27 to −1.91]; *p* = 0.009). No significant differences were found between CT, AT, and RT in altering body fat percentage (*p* > 0.05). For studies shorter than 10 weeks, no significant differences were noted across exercise modalities (*p* > 0.05). Under conditions where AT, RT, and CT workloads were matched, similar fat mass, body mass, body fat percentage, and FFM changes were observed between exercise modalities (*p* > 0.05). Similar body mass and body fat percentage loss was observed between same-day and different-day CT (*p* > 0.05); body fat mass loss only differed in a single study (*n* = 1) when comparing RT to different-day CT (aerobic and resistance exercises done on different days within a week).

**Conclusions:**

While there are no differences in percent body fat loss between exercise modes, AT and CT are more effective than RT alone in reducing absolute fat mass; however, RT neither improved nor impeded fat mass loss when incorporated into CT. Combining aerobic and resistance exercises on the same-day or different-day does not appear to influence the effectiveness of CT. When exercise interventions are short in duration (<10 weeks), there does not appear to be a difference in fat loss between exercise modalities. These results support the concurrent use of aerobic and resistance exercises for fat mass reduction, as well as an emphasis on workload and duration when programming exercise for fat loss.

## 1. Introduction

A combination of adequate nutrition and exercise is the recommended approach to reducing body fat, with both aerobic and resistance exercise modalities being purported as effective forms of exercise [1]. Concurrent training (CT), defined as the combination of resistance training (RT) and aerobic training (AT) within a single week of training [2], has been investigated for its effects on maximal strength, aerobic performance, and gains in fat-free mass (FFM) compared to AT and RT [2,3]. However, less attention has been given to body fat changes with CT [2,3].

Both AT and RT are suggested to augment fat loss by increasing energy expenditure and, by extension, inducing a state of negative energy balance. AT typically involves whole body repetitive and continuous exercise at a moderate-to-vigorous intensity [4]. RT directly impacts the musculoskeletal system, increasing skeletal muscle mass, which is an important site of metabolic activity [4]. However, it is not well understood if the combination of aerobic and resistance exercise can lead to a greater reduction in body fat than either strategy alone. With exercise modality being one of the main components of the FITT principle (frequency, intensity, time, and type) of exercising programming [4], an increased understanding of potential differing effects between exercise types may impact the choices that clinicians and fitness professionals make when programming exercise for patients and/or athletes. Specifically, more information is needed on how decisions that clinicians and fitness professionals can make regarding exercise programming can impact body fat loss outcomes, including the duration of their intervention, the timing of when aerobic and resistance exercises are included within a CT program, and if considerations should be made toward equating workloads between aerobic and resistance exercises during CT (i.e. “work-matched CT”).

To our knowledge, there have been three systematic reviews published that directly compared training adaptations between AT, RT, and CT [3,5,6]. Although, none of these systematic reviews included body fat changes as an outcome variable. Furthermore, one standalone meta-analysis on CT included a calculation of mean effect size changes in body fat mass; however, the inclusion criteria appeared to be limited to studies that reported measures of strength, power, and/or hypertrophy [7]. In addition, the authors did not report their search strategy for the inclusion/exclusion criteria [7].

The designs of previous systematic reviews have not allowed for the research question to be answered robustly. For these reasons, the primary purpose of this systematic review and meta-analysis was to examine studies from 1980 – when the earliest available and foundational study on CT was published [8] – through to 25 January 2023, that directly compared the effects of RT alone, AT alone, and CT on changes in body fat. To provide a comprehensive report on body composition changes, we also included studies that compared the effects of RT, AT, and CT on body mass and/or FFM. Additionally, we included sub-analyses focused on the other aspects of the FITT principle – frequency (when CT training sessions are programmed), intensity (whether workloads are matched between conditions), and time (length of exercise intervention). Lastly, potential biological sex differences were explored if sufficient data was available. Given the differing mechanisms behind aerobic and resistance exercises augmenting energy expenditure, we hypothesized that CT would differ in body fat changes compared to AT and RT alone regardless of intervention duration, CT timing, and work-matched CT protocols.

## 2. Methods

This systematic review and meta-analysis was conducted in accordance with the 2020 version of the Preferred Reporting Items for Systematic Reviews and Meta-Analyses (PRISMA) checklist [9]. Study inclusion criteria and methods were specified *a priori* and pre-registered on PROSPERO (CRD42023396530). Prior to data analysis, the decision was made to include sub-analysis comparisons based on work-matched CT, CT timing/scheduling (i.e. when AT and RT sessions were scheduled within a given week), and intervention duration. These sub-analyses were added to the PROSPERO protocol after pre-registration.

### 2.1. Search strategy

PubMed, SportDiscus, and Web of Science databases were searched to identify all relevant articles published up until 25 January 2023, with the following search concepts: “aerobic training,” “resistance training,” “concurrent training,” and “randomized controlled trial.” The reference lists of included studies were also searched for additional relevant articles. Unpublished research was not included in this systematic review and meta-analysis. The search string used for PubMed is available in Appendix A.

### 2.2. Identification and study selection

In line with the PICO (population, intervention, control, outcomes) acronym, all studies had to meet the following criteria to be included in the study: P) all participants had to be metabolically healthy adult humans (i.e. at least 18 years of age and free from metabolic diseases that would alter body composition, such as cancer or diabetes mellitus); I) the study had to be at least four weeks in duration, include a CT group in which aerobic and resistance exercises both occurred within the same week; C) the study had to be a randomized controlled trial in which a CT group was compared to an AT alone active control group (utilizing any form of aerobic exercise) and a RT alone active control group (utilizing free weights and/or weight machines); O) body mass, body fat mass and/or body fat percentage had to be reported at baseline and post-intervention. A secondary outcome measure of FFM was also extracted. If outcome variables were not reported as means ± standard deviation or standard error, the corresponding authors were contacted to obtain the data. In instances where those authors did not respond, the respective studies were included in the systematic review but not the meta-analysis. Studies that included animals, adolescents/children, or metabolically unhealthy participants were excluded from the systematic review and meta-analysis.

Database results were imported into a screening tool (Rayyan, Rayyan Systems, Cambridge, MA, USA). With the exceptions of AH, JC, and SF, all authors participated in title and abstract screening, which was completed in pairs. All authors were tested for inter-rater reliability prior to beginning screening using a subset of 10 studies. Percent agreement was calculated by determining the proportion of cases in which all raters agreed on their assessments out of the total number of cases evaluated. Across the 10 studies involving 110 individual assessments (10 studies × 11 raters), raters unanimously agreed on the correct responses in 109 cases, resulting in a percent agreement of approximately 99.1% [10]. In cases of disagreement within pairs, KL served as the tiebreaker. All reports that were deemed eligible or potentially eligible from the title and abstract screening had their full text screened to confirm eligibility. Full text screening was done by group consensus with the same authors that completed title and abstract screening. EndNote version 20 (Clarivate Analytics, London, UK) was used for all full-text screening.

### 2.3. Risk of bias assessment

Risk of bias due to study quality was assessed using the Physiotherapy Evidence Database (PEDro) scale [11]. The original PEDro scale consists of 11 dichotomous questions of which 10 are evaluated [11]. Scoring ranges from 0 to 10 where a higher score indicates a lower risk of bias [11]. Given that the assessors are rarely blinded, and that it is impossible to blind the participants in supervised exercise interventions, we elected to remove items 5–7 from the scale, which are specific to blinding. With the removal of these items, the maximum result on the modified PEDro 8-point scale was 7 (i.e. the first item is not included in the total score). The qualitative methodology ratings were adjusted similar to that used in previous exercise-related systematic reviews as follows: 6–7 = “excellent;” 5 = “good;” 4 = “moderate;” and 0–3 = “poor” [12,13]. Risk of bias was assessed by BIC and KL, with AR serving as a tiebreaker in cases of disagreement. No studies were excluded due to this quality assessment. Additionally, funnel plots were generated and visually inspected for publication bias (see Appendix B).

### 2.4. Data extraction

Data were extracted and organized into a Microsoft Excel spreadsheet (Version 2310, Microsoft Corporation, Redmond, WA, USA) independently by BIC and KL; any disagreements were reviewed and resolved by consensus with all authors, with the exceptions of AH, JC, and SF. Descriptive data regarding study design, as well as sample size, age, sex, and training status were extracted from each study. Baseline and post-intervention measures of body fat mass and body fat percentage were the primary outcome variables. Secondary outcome variables included body mass and FFM. All outcome variables were extracted from each study and separated by intervention group. Data was extracted as means ± standard deviation (SD) and/or error. If studies did not include data as means ± SD and/or error, their corresponding authors were contacted for the data.

### 2.5. Meta-analyses

Change scores were calculated by subtracting the pre-training mean from the post-training mean. SDs for the change scores were estimated from post and pre-training standard deviations using the following equation derived from the Cochrane Handbook for Systematic Reviews of Interventions: SD change Score = √[(SD baseline^2^ + SD final^2^) – (2 * correlation between baseline and final scores) * SD baseline * SD Final)] [14]. Standard errors were converted to standard deviations (SE = SD/√N). We used 0.8 as the correlation between final and baseline scores [15]. Heterogeneity was assessed using a chi-square test and the I^2^ statistic was used to quantify total variation across studies attributable to heterogeneity. Heterogeneity was determined by either a Chi^2^ p-value <0.01 or an I^2^ test value > 75%. Sensitivity analyses were conducted to assess the robustness of the overall estimates through exploring the effect of removing each individual study from the meta-analysis (“leave-one-out” analysis). We used random-effects models for our meta-analyses. Weighted mean differences (MD) and 95% confidence intervals were calculated. Forest plots were created using Review Manager Web 7.4.0 software (Cochrane Community, London, UK). Significance was established at *p* < 0.05.

Sub-analyses on intervention duration (<10 weeks versus ≥10 weeks), CT scheduling (aerobic and resistance exercises done on the same day versus different days within a week), work-matched CT, which compared studies that attempted to equate workloads between AT, RT, and CT, sex, and training status were considered if sufficient data was available.

## 3. Results

### 3.1. Study selection

**Figure 1.**
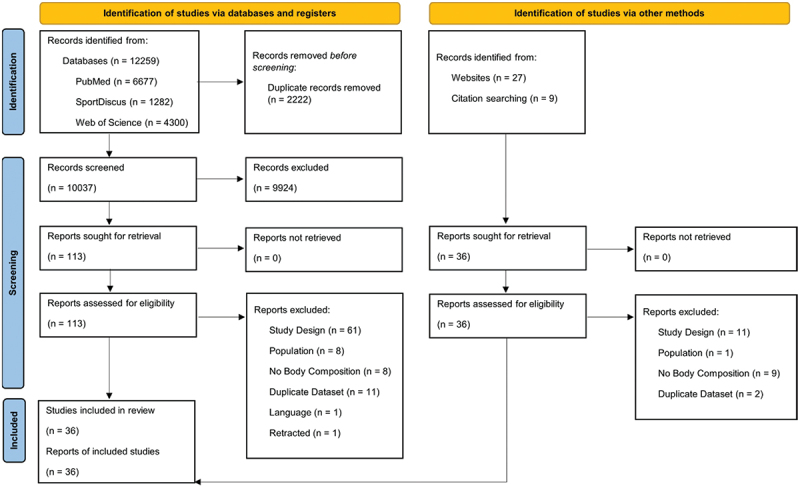
PRISMA flowchart of search results and screening [9].

Figure 1 provides a detailed overview of the study search and screening results. Of note, the earliest known publication on CT was excluded from this review and analysis as it was not a randomized controlled trial [8]. Two included reports originated from the same research study but analyzed different participants with no overlap and were therefore treated as two separate studies in our systematic review and meta-analysis [16,17]. One included report incorporated two different samples of older and younger adults [18], and therefore those two samples were treated as two separate studies in our systematic review and meta-analysis. Of the 36 studies included in the systematic review, only 31 studies were included in the meta-analysis [16–45].

The corresponding authors for studies that did not report data as means ± standard deviation or standard error at baseline and post-intervention were contacted for the data and followed up with up to two times over a six-week period. Three studies were excluded from meta-analyses due to no response from the corresponding authors [46–48]. One study was excluded from the meta-analysis due to no data being available [49], and another study was excluded from the meta-analysis due to no sample sizes being available [50]. Table 1 presents the study characteristics of all 36 included reports.

**Table 1.** Study characteristics of the aerobic, resistance, and concurrent training groups (N = 36).

Study	Analyzed Sample Size	Sex	Age (years)	BMI (kg/m^2^)	Body Mass (kg)	BF%	BF% Method	Training Status	Duration (weeks)	Resistance Training Type	Resistance Training Mode(s)	Aerobic Training Mode(s)	PEDro Scale Score
Ahtiainen 2009	26	M	50–67	>28	68.0–87.0	21–29	Skinfold	U	21	WB	WM/FW	Bike	7
Asad 2012	34	M	19.3–22.9	24.8–36.5	71.6–108.1	N/A	N/A	U	8	WB	WM/FW	Run	6
Ashutosh 1997	25	F	34–50	NR	78.3–114.5	N/A	N/A	U	52	NR	NR	Walk	4
Benito 2015	74	M/F	31.5–47.1	30.0–34.9	84.4–111.5	36.3–50.5	DEXA	U	22	WB	FW	Run, Bike, Elliptical	6
Benito 2020	66	M/F	29.3–45.5	25.0–29.9	68.9–91.4	30.05–48.0	DEXA	U	22	WB	FW	Run, Bike, Elliptical	6
Cadore 2010	23	M	61–69	NR	65.2–97.2	22.3–33.4	Skinfold	U	12	WB	WM/FW	Bike, Bike HIIT	5
Carnero 2014	45	F	29–48	24.5–33.7	64.4–86.6	34.5–48	DEXA	U	20	WB	WM	Walk, Bike	5
Chen 2017	45	M/F	65.8–73.3	23.0–32.7	53.4–81.5	33.9–45.5	BIA	U	8	WB	WM/FW	Step	5
Davidson 2009	93	M/F	61.4–75.6	25.5–34.2	70.1–99.5	33.1–48.3	MRI	U	24	WB	WM/FW	Walk	6
Dolezal 1998	30	M	18.5–21.7	NR	65.2–84.3	8.9–18.1	HW	T	10	WB	WM/FW	Run	7
Donges 2013	39	M	43.7–53.8	26.5–36.7	84.5–119.7	19.7–32.6	DEXA	U	12	WB	WM/FW	Bike, Elliptical	6
Dudley 1985	22	M/F	20.1–28.1	NR	47.3–80.8	N/A	N/A	U	7	LB	IK	Bike-Intervals	5
Ghahramanloo 2009	27	M	23–28	18.4–26.3	58.5–82.2	10.0–23.6	Skinfold	U	8	WB	WM/FW	Run	7
Glowacki 2004	41	M	20–30	NR	60.9–108.7	10.8–30.2	HW	U	13	WB	WM/FW	Run	6
Hennessy 1994	31	M	19.8–27.9	NR	68.6–91.6	11.9–24.3	Skinfold	T	8	WB	FW	Run	4
Ho 2012	48	M/F	43–64	23.4–45.6	71.3–107.8	28.8–55.5	DEXA	U	12	WB	FW	Run	6
Irving 2015 (Young)	34	M/F	18–30	20.7–30.5	57.3–95.4	24.4–43.9	DEXA	U	8	WB	WM/FW	Bike	6
Irving 2015 (Old)	30	M/F	66.8–77.9	23.8–31.3	62.4–94.6	29.7–46.3	DEXA	U	8	WB	WM/FW	Bike	7
Izquierdo 2005	31	M	40–46	NR	72.3–99.7	16.1–28.5	Skinfold	U	20	WB	WM/FW	Bike	6
Izquierdo 2004	31	M	62.2–69.9	16.5–33.7	67.2–92.6	17.2–29.7	Skinfold	U	20	WB	WM/FW	Bike	7
Kraemer 2004	34	M	20–29.4	NR	62.6–90.6	7.0–26.0	HW	U	12	WB	WM/FW	Run, Sprint Intervals	5
LeMura 2000	33	F	18–23	18.7–26.5	54.9–68.1	21.8–30.9	HW	U	16	WB	WM/FW	Bike, Row, Walk, Run	7
Libardi 2012	34	M	43.2–54.7	22.8–31.4	66.4–96.7	N/A	N/A	U	16	WB	WM	Walk, Run	6
Marks 1995	38	F	46.6–33.0	26.5–33.9	68.0–89.3	34.4–48.4	HW	U	20	WB	WM	Bike Intervals	4
McCarthy 1995	30	M	24.9–29.1	NR	77.6–90.0	16.8–22.7	Skinfold	U	10	WB	WM/FW	Bike	6
Nelson 1990	14	M	23.1–34.8	NR	61.0–100.8	5.4–29.5	HW	U	20	LB	IK	Bike	4
Ramirez-Velez 2020	41	M/F	33.7–47.8	26.6–33.5	64.9–96.5	33.2–44.9	DEXA	U	12	WB	FW	Run-HIIT	3
Shakiba 2019	33	M	NR	26.6–34.0	77.7–103.8	N/A	N/A	U	12	WB	FW	Run-Intervals	6
Shaw 2011	38	M	19.4–30.6	19.7–31.8	60.6–97.8	N/A	N/A	U	16	WB	NR	Bike, Row, Walk, Step	5
Sillanpaa 2010	70	F	39–64	21.5–27.7	56–78	27.9–42.9	DEXA	U	21	WB	WM	Bike	6
Sillanpaa 2008	42	M	40–65	21.7–27.7	63.5–90.5	18.2–33.5	DEXA	U	21	WB	WM	Bike	5
Sillanpaa 2009	50	F	39–64	20.3–26.0	52.0–70.4	24.8–40.3	DEXA	T	21	WB	WM	Bike	5
Timmons 2018	63	M/F	66.1–74.5	20.9–31.2	56.5–97.9	24.4–41.4	DEXA	U	12	WB	FW	Bike, Elliptical	5
Tseng 2013	30	M	18–29	29.3–32.4	76.9–109.6	N/A	N/A	U	12	WB	WM	Walk	5
Villareal 2017	120	M/F	65–75	31.3–42.5	80.9–120.3	NR	DEXA	U	26	WB	WM	Walk, Bike, Stairs	6
Wadden 1997	99	F	30.9–51.1	30.5–42.4	82.6–109.6	38.9–53.6	HW	U	48	WB	WM	Step	4

Ranges for age, BMI, body mass, and body fat percentage were estimated by ± 1 standard deviation from the baseline means of the aerobic, resistance, and concurrent training groups if the range was not reported.

Abbreviations: BF% = body fat percentage; BIA = bioelectrical impedance analysis; BMI = body mass index; DEXA = dual energy x-ray absorptiometry; F = female participants; FW = free weights; HIIT = high-intensity interval training; HW = hydrostatic weighing; IK = iso-kinetic dynamometry; LB = lower-body; *M* = male participants; MRI = magnetic resonance imaging; N/A = not assessed; NR = not reported; *T* = trained participants; U = untrained participants; WB = whole-body; WM = weight machines.

### 3.2. Study quality assessment

Study quality was assessed using a modified PEDro scale with scores ranging from 0–7. Only 1 study included in this systematic review and meta-analysis was considered “poor quality” [39], and all other included studies were at least of “moderate quality.”

### 3.3. Meta-analyses (overall effects)

**Figure 2.**
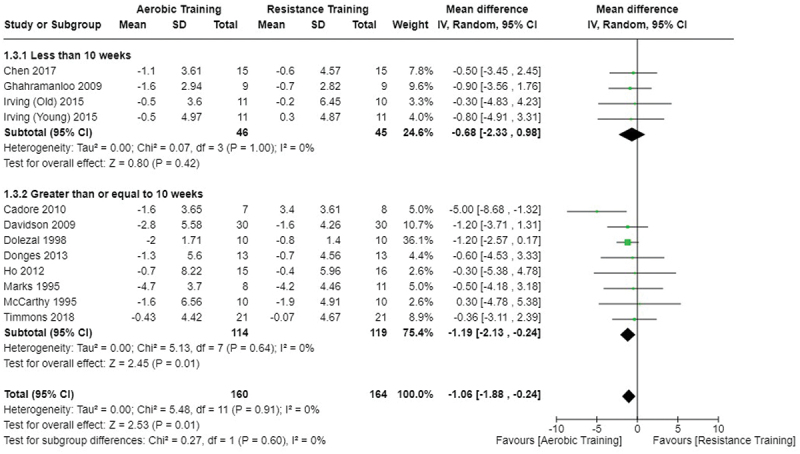
Comparison of aerobic and resistance training for fat mass loss. Means, standard deviations (SD), and 95% confidence intervals (CI) are presented in kg.

**Figure 3.**
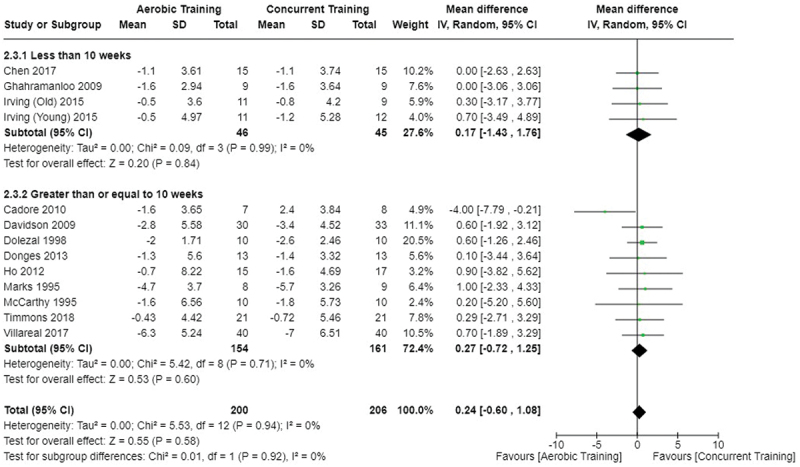
Comparison of aerobic and concurrent training for fat mass loss. Means, standard deviations (SD), and 95% confidence intervals (CI) are presented in kg.

**Figure 4.**
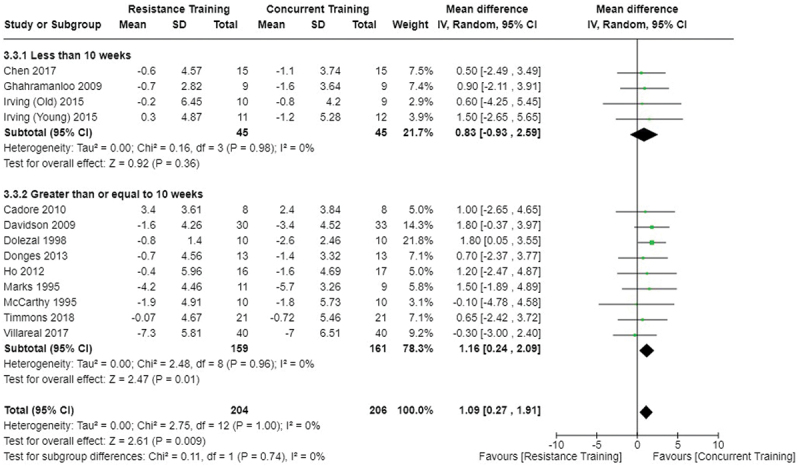
Comparison of resistance and concurrent training for fat mass loss. Means, standard deviations (SD), and 95% confidence intervals (CI) are presented in kg.

Figures 2–4 present the comparisons between AT, RT, and CT regarding fat mass changes. AT and CT did not differ in fat mass changes; however, both AT and CT were favored over RT for fat mass loss.

**Figure 5.**
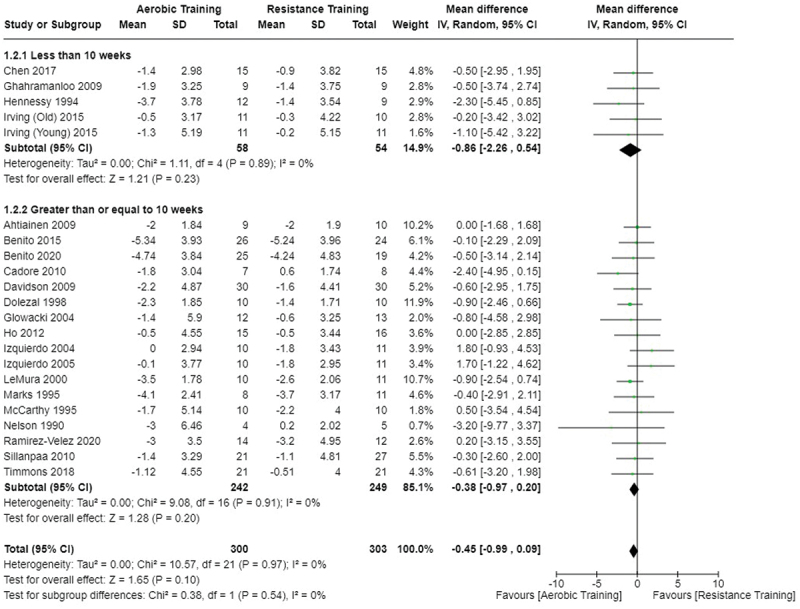
Comparison of aerobic and resistance training for body fat percentage loss. Means, standard deviations (SD), and 95% confidence intervals (CI) are presented in kg.

**Figure 6.**
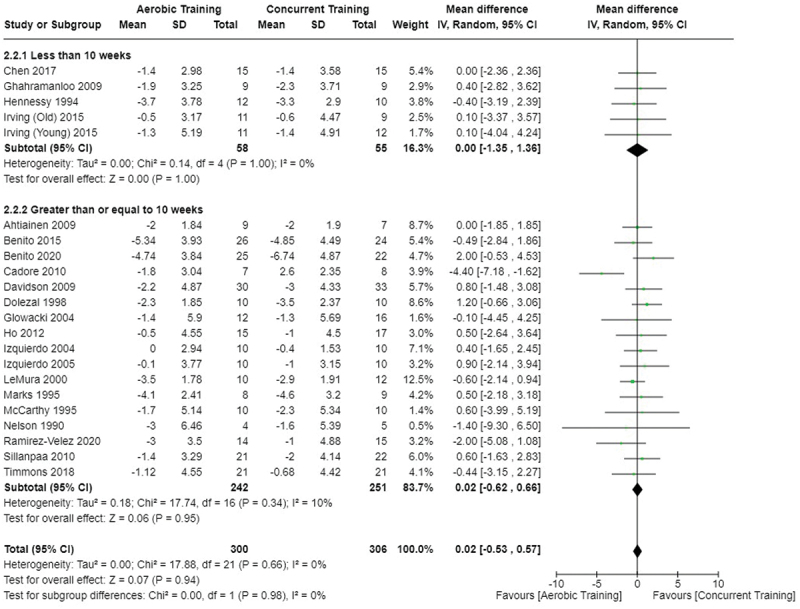
Comparison of aerobic and concurrent training for body fat percentage loss. Means, standard deviations (SD), and 95% confidence intervals (CI) are presented in kg.

**Figure 7.**
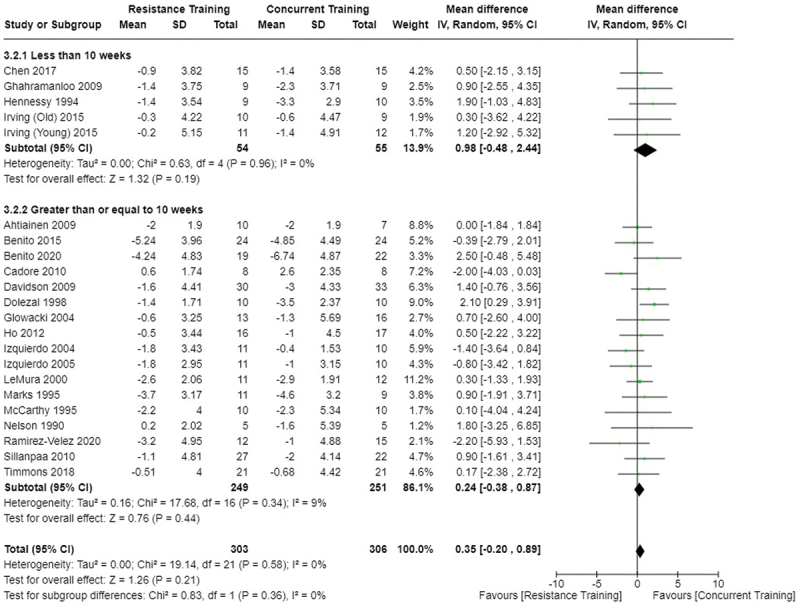
Comparison of resistance and concurrent training for body fat percentage loss. Means, standard deviations (SD), and 95% confidence intervals (CI) are presented in kg.

Figures 5–7 present the comparisons between AT, RT, and CT regarding body fat percentage changes. AT, RT, and CT did not differ in body fat percentage changes.

**Figure 8.**
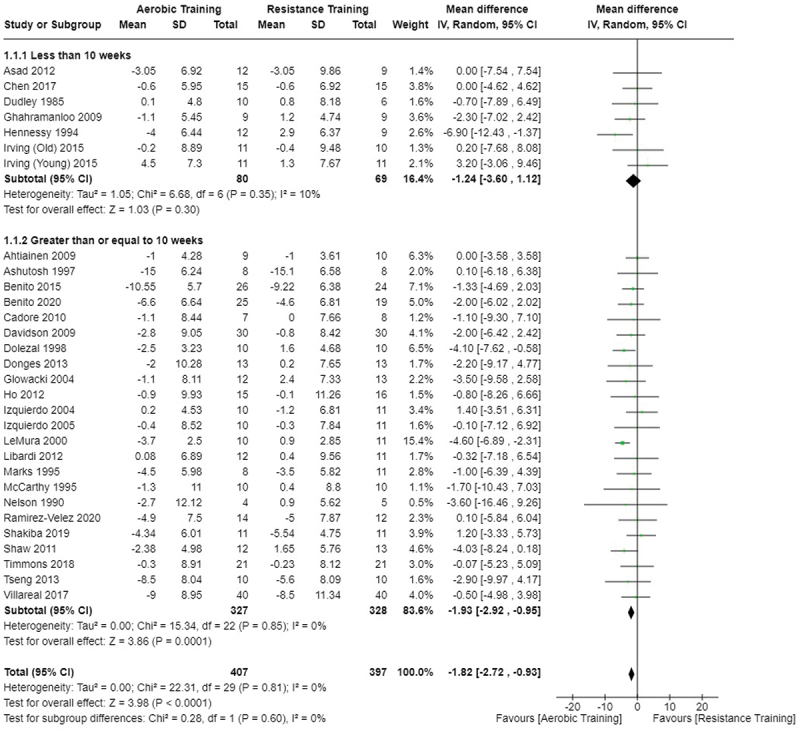
Comparison of aerobic and resistance training for body mass loss. Means, standard deviations (SD), and 95% confidence intervals (CI) are presented in kg.

**Figure 9.**
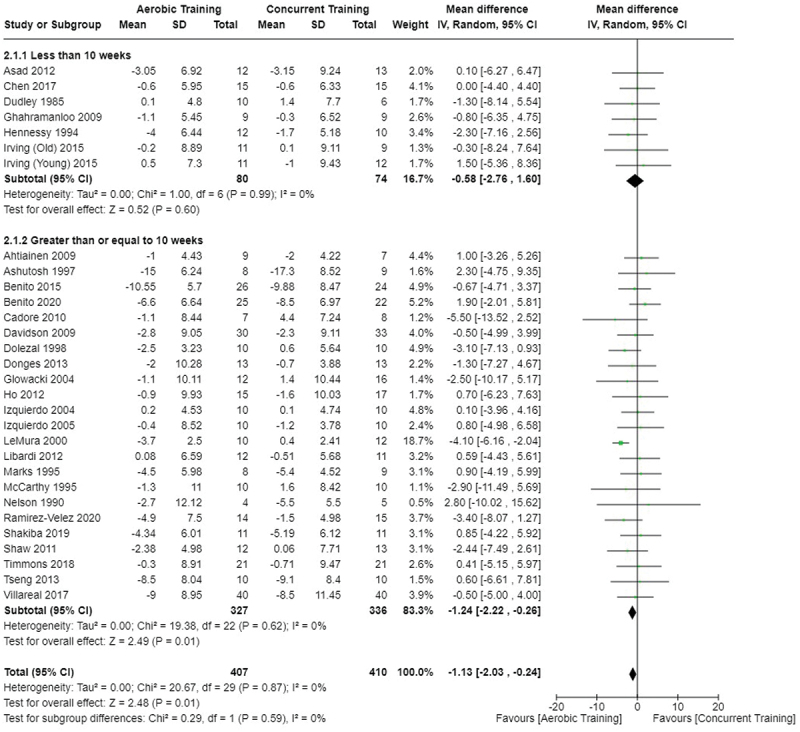
Comparison of aerobic and concurrent training for body mass loss. Means, standard deviations (SD), and 95% confidence intervals (CI) are presented in kg.

**Figure 10.**
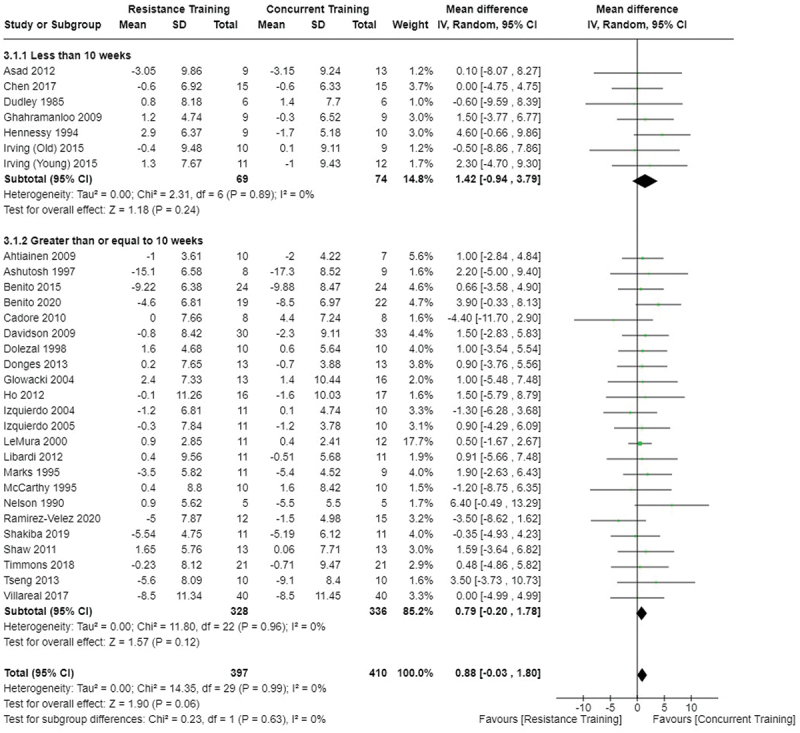
Comparison of resistance and concurrent training for body mass loss. Means, standard deviations (SD), and 95% confidence intervals (CI) are presented in kg.

Figures 8–10 present the comparisons between AT, RT, and CT regarding body mass changes. AT provided significantly greater body mass loss compared to RT and CT. No differences in body mass changes were observed between RT and CT.

**Figure 11.**
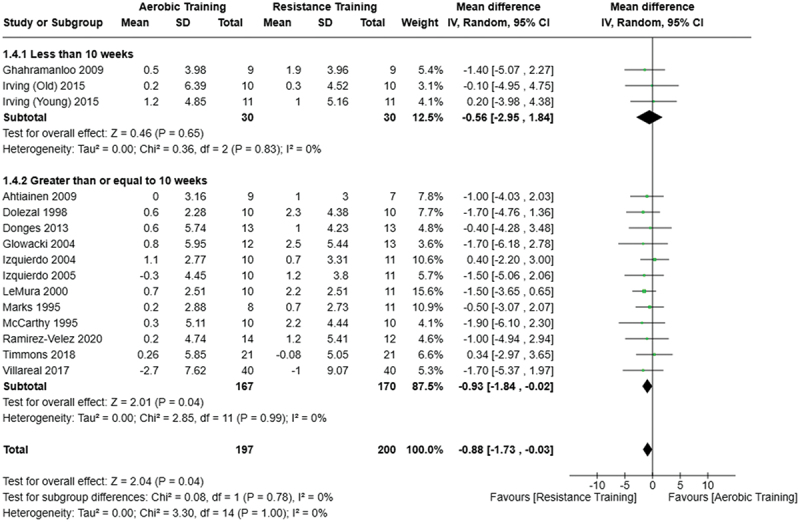
Comparison of aerobic and resistance training for fat-free mass changes. Means, standard deviations (SD), and 95% confidence intervals (CI) are presented in kg.

**Figure 12.**
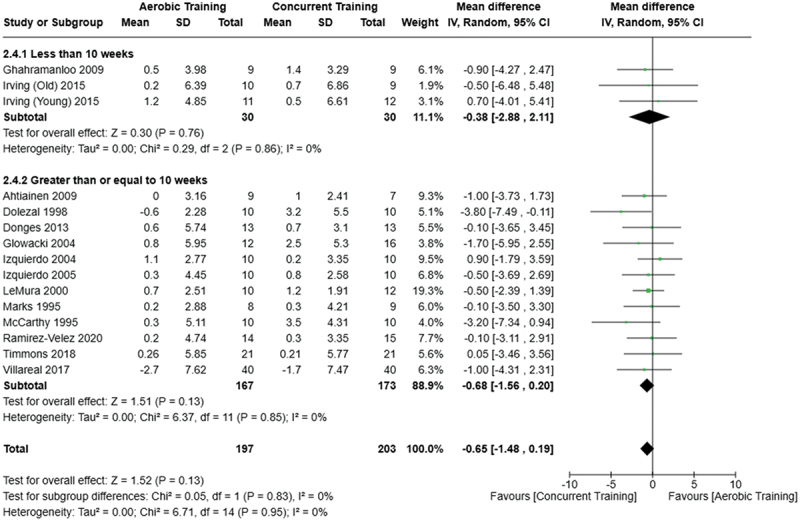
Comparison of aerobic and concurrent training for fat-free mass changes. Means, standard deviations (SD), and 95% confidence intervals (CI) are presented in kg.

**Figure 13.**
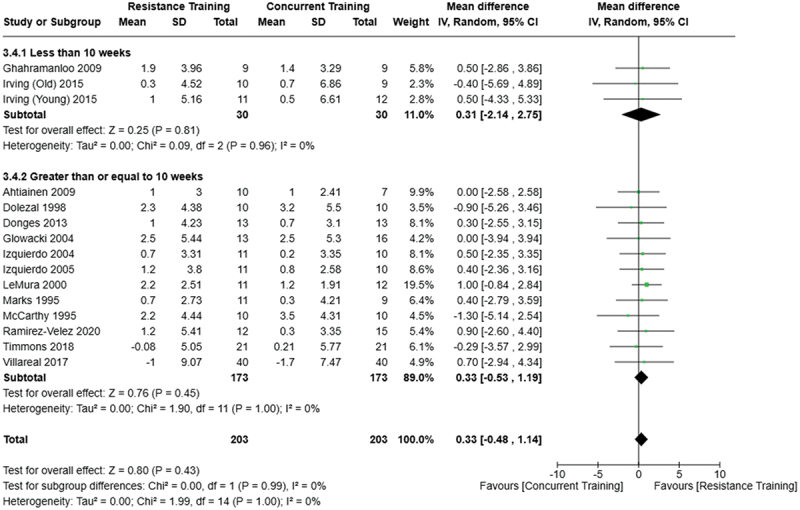
Comparison of resistance and concurrent training for fat-free mass changes. Means, standard deviations (SD), and 95% confidence intervals (CI) are presented in kg.

Figures 11–13 present the comparisons between AT, RT, and CT regarding FFM changes. CT provided similar FFM changes when compared to AT and RT. However, a greater increase in FFM was observed with RT compared to AT.

To assess the robustness of the meta-analyses, a leave-one-out analysis was performed. For the RT vs. AT comparison for FFM, results went from significant to non-significant with the removal of Ghahramanloo 2009 [28], Dolezal 1998 [25], Izquierdo 2005 [31], LeMura 2000 [33], McCarthy 1995 [37], and Villareal 2017 [45]. For the AT vs. CT comparison for body mass, removal of LeMura 2000 [33] altered the findings. For the CT vs. RT comparison for body mass, removal of Chen 2017 [23], Dudley 1985 [27], Irving 2015 (old) [18], Cadore 2010 [22], Izquierdo 2004 [32], McCarthy 1995 [37], Ramirez-Velez 2020 [39], Shakiba 2019 [40], and Villareal 2017 [45], all altered the findings from being non-significant to significant. Furthermore, for the CT and RT comparison for fat mass, removal of Dolezal 1998 [25] altered the findings. All other comparisons were not influenced by the removal of any individual study.

### 3.4. Sub-analyses

Data for intervention duration sub-analyses are presented in Figures 2–13. AT, RT, and CT did not differ in fat mass, body fat percentage, body mass, or FFM changes with short-duration (<10 weeks) interventions. Longer duration interventions (≥10 weeks) provided identical effects as the previously listed overall effects.

**Figure 14.**
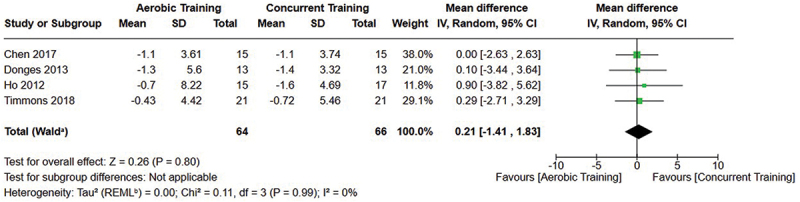
Comparison of aerobic and work-matched concurrent training for fat mass loss. Means, standard deviations (SD), and 95% confidence intervals (CI) are presented in kg.

**Figure 15.**
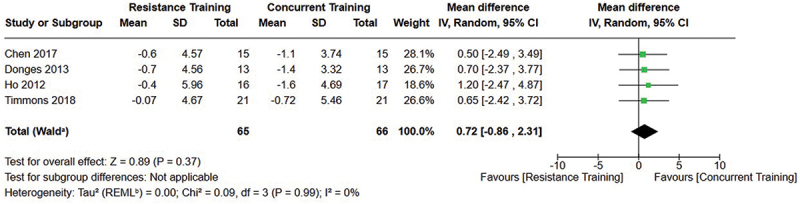
Comparison of resistance and work-matched concurrent training for fat mass loss. Means, standard deviations (SD), and 95% confidence intervals (CI) are presented in kg.

**Figure 16.**
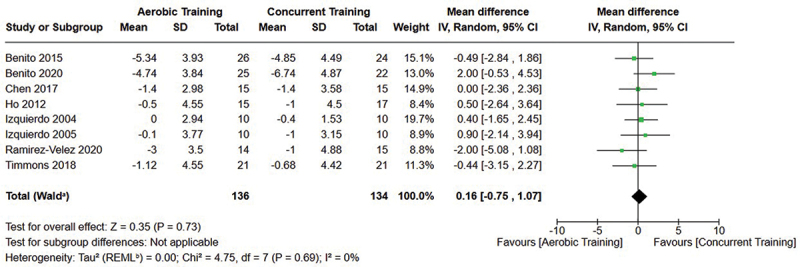
Comparison of aerobic and work-matched concurrent training for body fat percentage loss. Means, standard deviations (SD), and 95% confidence intervals (CI) are presented in %.

**Figure 17.**
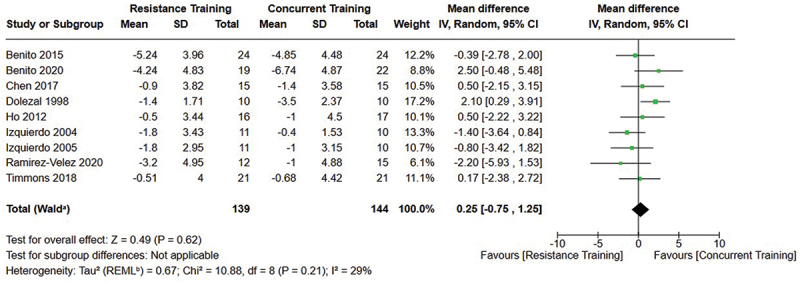
Comparison of resistance and work-matched concurrent training for body fat percentage loss. Means, standard deviations (SD), and 95% confidence intervals (CI) are presented in %.

**Figure 18.**
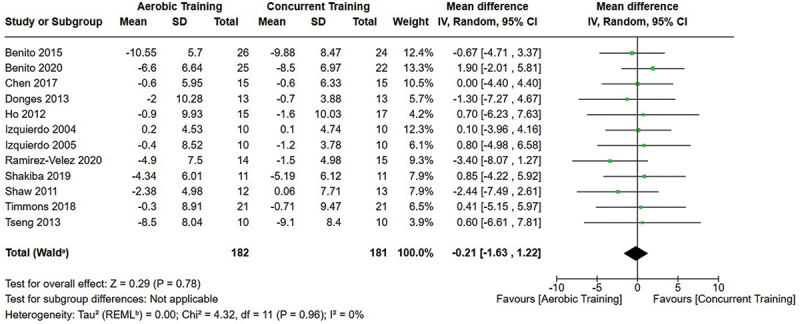
Comparison of aerobic and work-matched concurrent training for body mass loss. Means, standard deviations (SD), and 95% confidence intervals (CI) are presented in kg.

**Figure 19.**
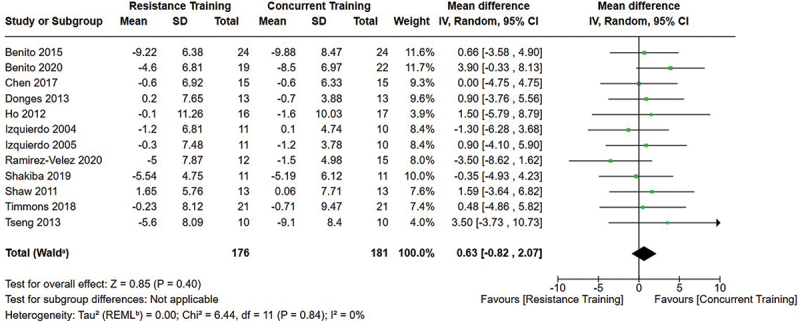
Comparison of resistance and work-matched concurrent training for body mass loss. Means, standard deviations (SD), and 95% confidence intervals (CI) are presented in kg.

**Figure 20.**
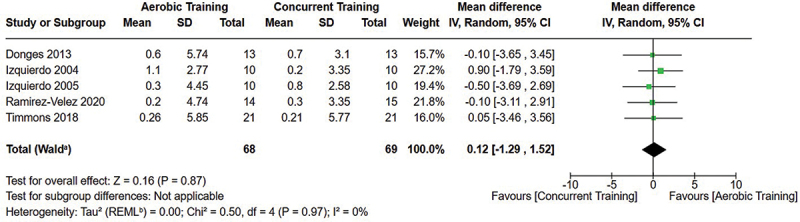
Comparison of aerobic and work-matched concurrent training for fat-free mass changes. Means, standard deviations (SD), and 95% confidence intervals (CI) are presented in kg.

**Figure 21.**
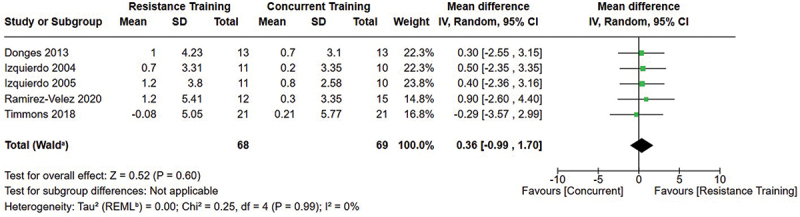
Comparison of resistance and work-matched concurrent training for fat-free mass changes. Means, standard deviations (SD), and 95% confidence intervals (CI) are presented in kg.

Figures 14–21 provide the results of our work-matched CT sub-analysis on each outcome variable. Only 12 studies from our original sample attempted to equate workloads between conditions. Nine studies did so by controlling the duration of each workout session [23,26,30–32,40,41,43,44]. Two studies attempted to equate volume assessed via heart rate reserve for aerobic exercise and percentage of 15-repetition maximum for resistance exercises [16,17]. One study attempted to equate volume by calculating work measured in joules using heart rate for aerobic exercises and repetition volume for resistance exercises [39]. No differences in body mass, fat mass, body fat percentage, nor FFM changes were observed between AT, RT, and work-matched CT (Figures 14–21).

**Figure 22.**
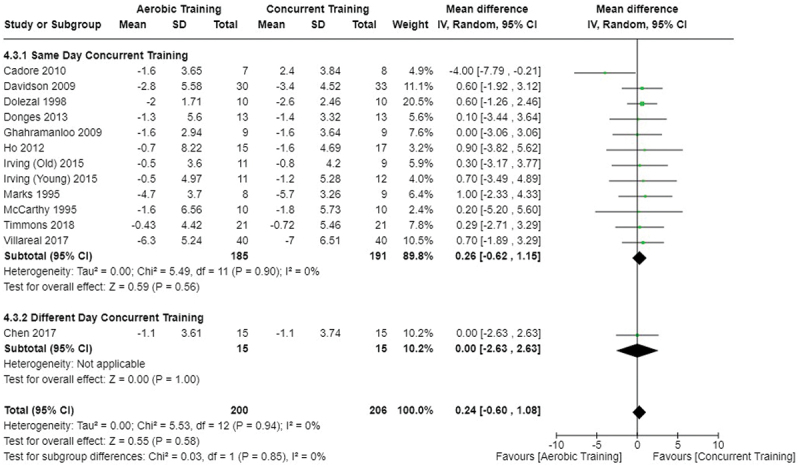
Comparison of aerobic and same/different day concurrent training for fat mass loss. Means, standard deviations (SD), and 95% confidence intervals (CI) are presented in kg.

**Figure 23.**
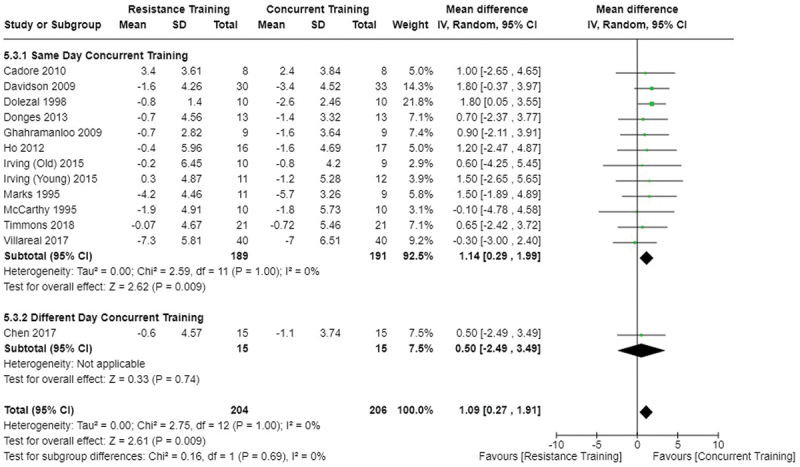
Comparison of resistance and same/different day concurrent training for fat mass loss. Means, standard deviations (SD), and 95% confidence intervals (CI) are presented in kg.

**Figure 24.**
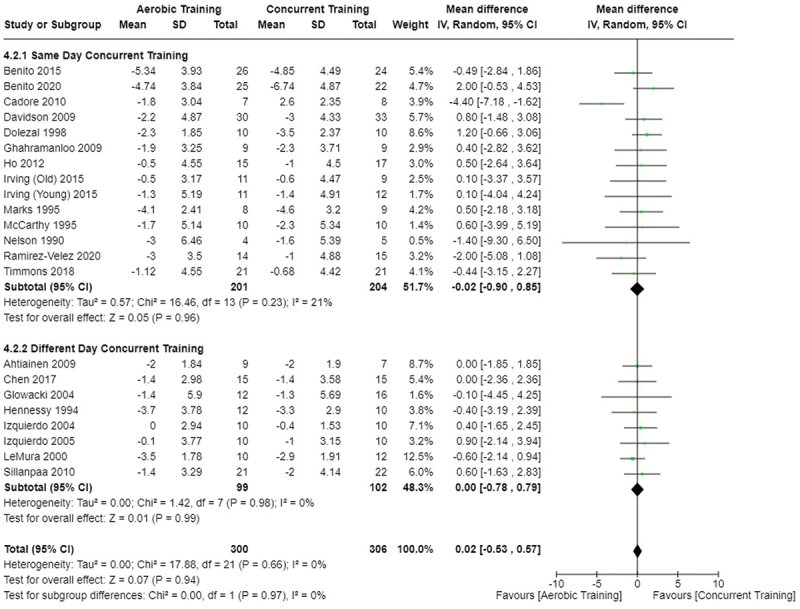
Comparison of aerobic and same/different day concurrent training for body fat percentage loss. Means, standard deviations (SD), and 95% confidence intervals (CI) are presented in %.

**Figure 25.**
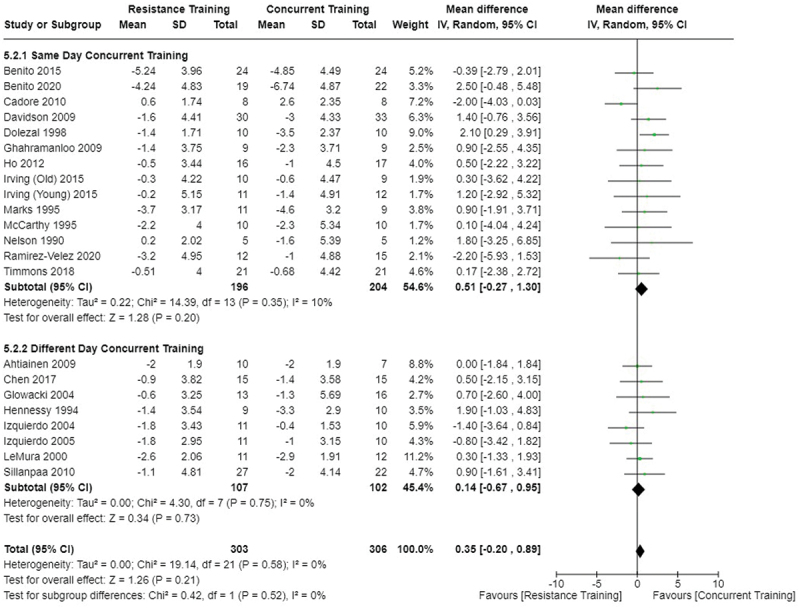
Comparison of resistance and same/different day concurrent training for body fat percentage loss. Means, standard deviations (SD), and 95% confidence intervals (CI) are presented in %.

**Figure 26.**
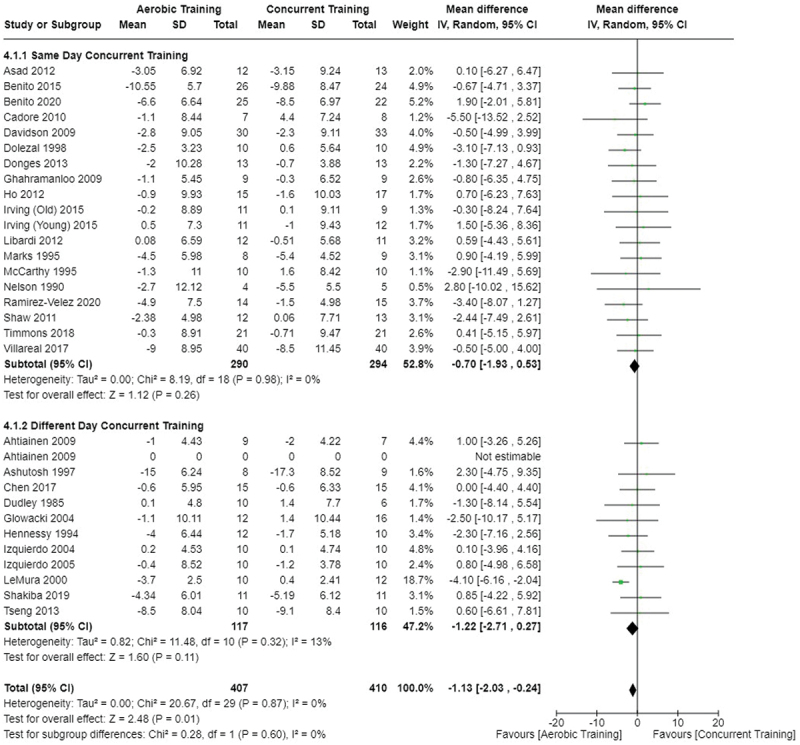
Comparison of aerobic and same/different day concurrent training for body mass loss. Means, standard deviations (SD), and 95% confidence intervals (CI) are presented in kg.

**Figure 27.**
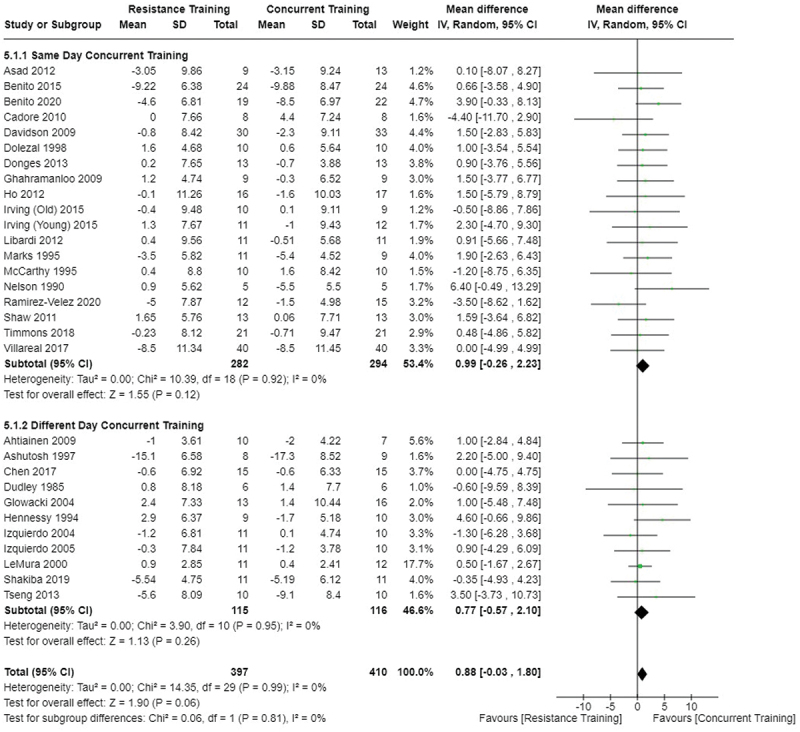
Comparison of resistance and same/different day concurrent training for body mass loss. Means, standard deviations (SD), and 95% confidence intervals (CI) are presented in kg.

**Figure 28.**
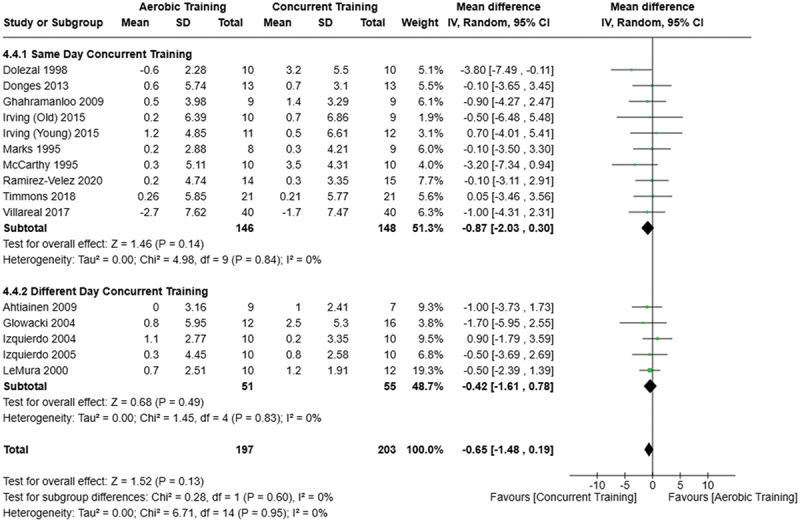
Comparison of aerobic and same/different day concurrent training for fat-free mass changes. Means, standard deviations (SD), and 95% confidence intervals (CI) are presented in kg.

**Figure 29.**
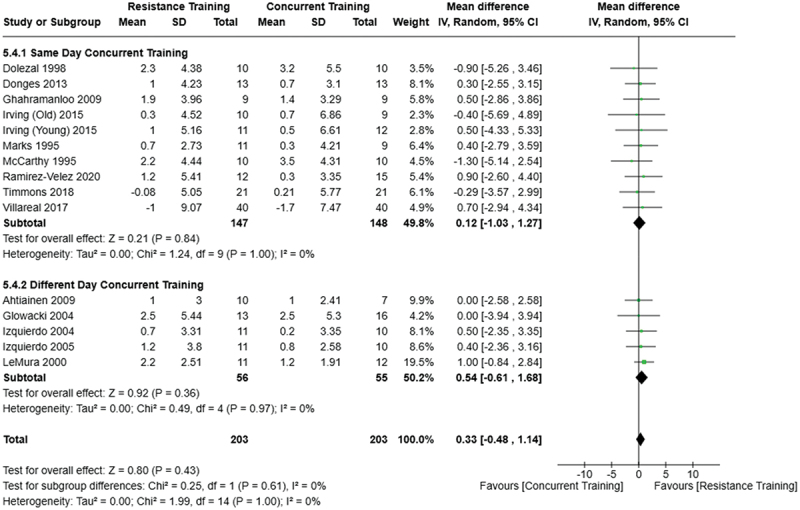
Comparison of resistance and same/different day concurrent training for fat-free mass changes. Means, standard deviations (SD), and 95% confidence intervals (CI) are presented in kg.

Figures 22–29 provide the results of our sub-analysis on CT timing (i.e. scheduling AT and RT on the same or different days in a given week) for each outcome variable. Comparing RT to CT, there was a significant difference in fat mass loss, for interventions ≥10 weeks and overall, favoring same-day CT but not different-day CT (Figure 23). No other comparisons between same-day and different-day CT were statistically significant (Figures 22–29).

There were only 3 studies that used trained participants [25,34,47], as such there was insufficient data to conduct the training status sub-analysis. There were seven studies that used only female participants [21,33,36,47–49], and there were no significant differences between exercise modes for body mass, fat mass, % body fat, or FFM (*p* > 0.05). There was a non-significant trend for body mass loss to be greater following AT compared to RT (MD: −2.93 kg [95% CI = −5.91 to 0.05]; *p* = 0.05).

## 4. Discussion

This study compared the effects of AT, RT, and CT on fat mass, body fat percentage, body mass, and FFM in metabolically healthy adults. This systematic review and meta-analysis included 36 randomized controlled trials, including a total of 1564 research participants, spanning from January 1980 to January 2023. Our findings suggest that, compared to RT, AT leads to significantly greater loss of body mass (MD = −1.83 kg, *p* < 0.001) and fat mass (MD = −1.06 kg, *p* = 0.01) while also preserving less FFM (MD = −0.88 kg, *p* = 0.04). Compared to CT, AT appears to result in significantly greater body mass loss (MD = −1.13 kg, *p* = 0.01) but no difference in body fat percentage, fat mass, or FFM changes (*p* > 0.05). Additionally, when CT is juxtaposed with RT, a statistically significant difference in fat mass reduction is observed, favoring CT (MD = 1.09 kg, *p* = 0.009); yet no statistically significant differences are noted in body mass loss, body fat percentage loss, or FFM retention (*p* > 0.05). For interventions lasting less than 10 weeks, no differences were observed in any body composition changes (*p* > 0.05). Furthermore, in sub-analyses focusing on work-matched CT versus RT and AT, no statistically significant differences in body mass, body fat percentage, fat mass, nor FFM changes were observed (Figures 14–21). This suggests that when adjusted for similar energy expenditure, CT provides similar body composition outcomes as AT or RT alone. Additionally, same-day CT presented statistically significant improvements in fat mass loss when compared to RT (MD = 1.14 kg, *p* = 0.009). No significant differences were observed when comparing AT or RT to different-day CT.

The results suggest that, when not attempting to match work volume, both AT and CT are favored over RT for fat mass loss. Comparing AT with CT there was no significant difference in fat mass loss (Figure 3), suggesting that combining both AT and RT neither hinders nor enhances the fat loss process. However, compared to CT, AT led to significantly greater body mass loss with no difference in body fat percentage loss. Given a two-compartment body composition model, greater body mass loss with no difference in body fat percentage loss would logically be explained by FFM retention; however, CT was statistically similar in FFM changes compared to AT while AT preserved FFM less than RT alone (Figures 11–13). Both RT and CT include resistance exercises, which are understood to help preserve FFM during caloric deficits through an upregulation of muscle protein synthesis [51]. Beyond the two-compartment model, FFM also includes retained water and stored glycogen which may be modified by AT and lead to similar FFM changes as CT. The inclusion of both aerobic and resistance exercises within CT appears to have placed it in the middle of AT and RT, where CT does not statistically differ in FFM retention from AT nor RT, yet RT provides superior FFM compared to AT. There may potentially be practically, but not statistically, significant differences between CT and other modalities for FFM retention which should be considered by coaches/trainers and clinicians aiming to preserve FFM loss. Additionally, more research is needed utilizing four- or five-compartment models of body composition to better understand how CT, AT alone, and RT alone modify FFM and if an interference effect is present.

To the best of our knowledge, this is the first meta-analysis to directly compare AT to CT and RT for whole-body FFM changes. Previous systematic reviews have compared CT to RT for muscle hypertrophy, both reporting no differences in line with our findings [3,6]. This is despite methodological differences, as both Sabag et al. [6] and Schumann et al. [3] included studies that examined regional hypertrophy rather than whole body, and they focused on muscular hypertrophy rather than whole-body FFM. Nonetheless, the present results indicate that the inclusion of resistance exercises in a training regimen neither impedes nor enhances fat loss, and that AT (not CT) is disadvantageous for preserving FFM compared to RT alone but also advantageous for achieving body mass loss in healthy adults. The greater body mass loss in AT is likely attributed to a higher energy expenditure during each exercise session [52]. In comparison, RT and CT both include rest periods which could potentially result in less work being done (i.e. energy expenditure) during each exercise session compared to continuous AT. Therefore, this meta-analysis included a sub-analysis of studies that attempted to equate workload (i.e. work-matched).

When studies attempted to equate duration or estimated caloric expenditure between exercise modalities, there were no observed differences in fat mass, body fat percentage, body mass, or FFM changes. This supports the previously mentioned notion that, without work-matching, AT provides superior body mass loss via increased energy expenditure compared to RT and CT, as both latter modalities involve rest periods. Despite the use of rest periods reducing the total energy expenditure during each exercise session (assuming equal session duration), RT was traditionally understood to increase energy expenditure after each exercise session via excess post-oxygen consumption (EPOC) [53,54]. However, recent evidence has shown that increases in EPOC are dependent on intensity rather than exercise modality, with interval and continuous AT eliciting similar EPOC responses compared to RT when workloads were matched between groups [55,56]. Similar energy expenditure and EPOC with work-matched protocols likely explain the observed similarities in both body mass and body fat loss. This allows for preference in exercise selection for those desiring body fat or body mass loss. However, if an increase in FFM is desired, then coaches/clinicians should ensure that RT is included in training protocols regardless of energy expenditure, with RT alone appearing to provide superior FFM retention than AT (Figure 11). Additionally, due to the inclusion of rest periods in RT and CT, aiming to increase energy expenditure via these modalities may necessitate an increase in training session duration to achieve a similar caloric expenditure as continuous AT [52]. Alternatively, techniques such as supersets and circuits may be used to mitigate the need for extended training durations [57,58].

While the present results indicate no differences in body fat and body mass loss between exercise modalities when the workload is equated, our findings should be interpreted cautiously. The requirement of workload matching for inclusion in this sub-analysis led to a reduced number of studies, and the included studies varied in their approach to matching workloads. Despite these limitations, the work-matched sub-analyses highlight the importance of considering the impact of workloads when comparing different exercise modalities in the context of body mass loss and body composition changes. Future research should look to match workload through caloric expenditure measured via indirect or direct calorimetry.

Regarding our sub-analysis examining study durations shorter than 10 weeks, no significant differences in body mass, body fat percentage, fat mass, nor FFM changes were observed between AT, RT, and CT. Further, the comparisons between AT, RT, and CT within studies lasting at least 10 weeks in duration were identical to the overall meta-analysis effects for all analyses. While the dose-response relationship between exercise frequency and duration during weight loss interventions are well understood [59], there is limited evidence to suggest that a particular mode of exercise confers greater fat and body mass loss in shorter duration interventions. Despite the limited evidence, AT typically is utilized by individuals looking to reduce fat mass and body mass during short interventions such as a bodybuilding preparation phase or a combat athlete fight camp [60–62]. These results provide evidence for clinicians and coaches to support the prioritization of other factors such as athlete/patient preference instead of body composition changes when determining exercise modality for short-term interventions (<10 weeks) given the similar body composition changes between AT, RT, and CT for short-term interventions. However, these results are limited to a scope of chronic adaptations, as the shortest duration for an included study was 7 weeks. For some populations, such as athletes in sports with weight categories, there may be greater need to modify body composition sub-acutely (i.e. within several days) via exercise training [62], but more research is warranted on potential differences in exercise modality efficacy for sub-acute changes. Overall, these results highlight the importance of intervention duration to elicit meaningful changes in body composition, as duration is one of the modifiable factors within the controls of exercise programming.

Exercise timing/scheduling is another key modifiable factor for exercise programming, especially for CT as both resistance and aerobic exercise sessions must be programmed together within a given week [2]. A sub-analysis was conducted comparing same-day CT and different-day CT protocols against RT and AT. Interestingly, the only observed significant difference was between RT and CT regarding fat mass loss (Figure 23) where same-day CT led to significantly greater fat mass loss compared to RT, but fat mass loss was similar between different-day CT and RT. However, this sub-analysis was limited in sample size and power, where only 1 study compared different-day CT to RT and AT [23]. Considering the limitation, these results suggest that there is no meaningful effect of aerobic and resistance exercise scheduling on the overall efficacy of CT for body mass and fat loss. This would support the use of personal preference when determining the timing of resistance and aerobic exercises in a CT program.

The present systematic review and meta-analysis is not without limitations. The included studies utilize a range of validated body composition techniques, which vary in standard error of measurement and estimation. This is consistent with previous systematic reviews on body composition changes [3,6]. Furthermore, variations in error exist within single techniques (e.g. variations between different models of BIA devices) [63], and the included body composition techniques are understood to be valid and reliable within 5% of total error [64]. Aside from error in the assessment of body composition, potential age and sex related differences may impact the observed results of the meta-analysis. Previous research has suggested that older adults ≥60 years of age may experience weight loss at a greater rate than younger adults [65,66]. However, methodological limitations to those studies include the inclusion of only high-responders and quasi-experimental designs [65,66]. Currently, there is a lack of compelling evidence to warrant an age-based sub-analysis, as more research is needed directly comparing exercise-induced body composition changes between older and younger adults. Likewise, previous research on relative body composition changes to weight loss interventions between sexes are equivocal [67,68]. While we did conduct sex-based sub-analyses (Appendix C), the results should be interpreted with caution as they included no more than 4 studies, are likely not robust enough to be meaningful, and were therefore not included directly in this manuscript. While there is likely a plethora of sub-analyses that could be completed, we chose to focus on the most externally valid sub-analyses with direct practical application to exercise program design decisions through the FITT principle, such as intervention duration, workout timing/frequency, and workload management, as a single study cannot feasibly account for all possible sub-analyses. Additionally, we were unable to control for potential differences in dietary habits, including the use of dietary supplements, due to inconsistent reporting between the included studies. However, the studies that did report specific dietary constraints within their study design (e.g. a 25% caloric deficit) had all groups undergo the same dietary constraint [16,17,21,24,36,39], as all included studies were designed to investigate differences between AT, RT, and CT for body composition changes. This systematic review and meta-analysis are among the first to directly compare body composition changes from AT, RT, and CT, thereby providing clinicians and fitness professionals with practical findings to apply to their clinical and coaching practices regarding how they program exercise for those pursuing fat loss and/or weight loss.

## 5. Conclusion

Both AT and CT appear to be more favorable for fat mass loss compared to RT. The absence of significant differences between AT and CT in fat mass loss suggests that combining aerobic and resistance exercises does not hinder or improve the fat loss process. However, our findings suggest that RT alone results in superior FFM retention compared to AT alone, but not CT which does not statistically differ in FFM preservation from AT or RT. There may be a practical, but not statistical, difference in FFM preservation with CT compared to RT due to the inclusion of aerobic exercises (i.e. a potential interference effect), but more research utilizing robust measures of body composition and workloads is needed to confirm that theory.

The importance of workload matching is highlighted in the sub-analyses, where studies attempting to equate duration or caloric expenditure between modalities showed no differences in body mass, body fat percentage, fat mass, and FFM changes. This underscores the significance of considering workload when comparing different exercise modalities in the context of body mass loss and body composition changes. Nevertheless, there is a need for cautious interpretation, as the requirement for workload matching in the sub-analysis resulted in a reduced sample size of studies with variations in their approach to matching workloads. Future research in this area should attempt to match workloads through methods such as calorimetry to ensure a robust study design and contribute further to our understanding of the nuanced effects of different exercise modalities on body composition.

## Supplementary Material

Supplemental Material
