# Supplementary material for: Comparison of concurrent, resistance, or aerobic training on body fat loss: a systematic review and meta-analysis
Source: J Int Soc Sports Nutr. 2025 May 22;22(1):2507949. doi: 10.1080/15502783.2025.2507949 (PMC12107660; doi:10.1080/15502783.2025.2507949)
Supplement: Supplemental Material [file RSSN_A_2507949_SM0656.zip › Supp/CRAB Study Appendix B_RevisedSubmission.docx]

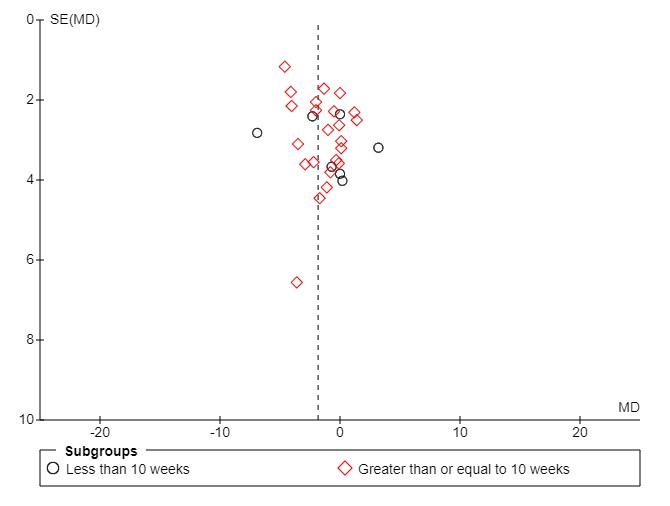


Figure S1B - Resistance Training vs. Aerobic Training for Weight Loss


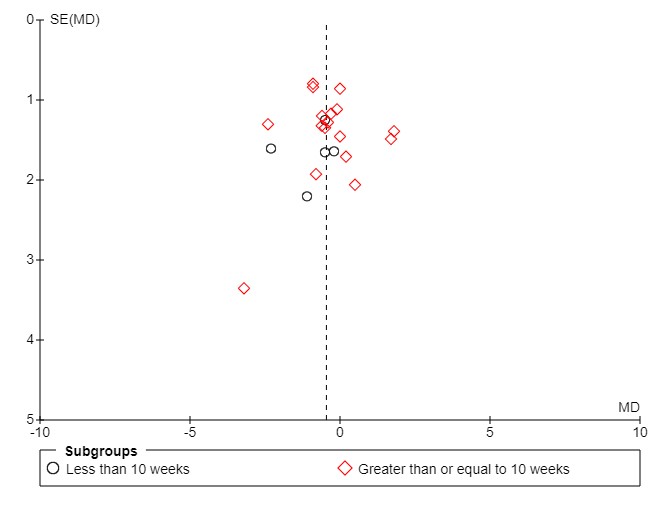


Figure S2B - Resistance Training vs. Aerobic Training for % Body Fat


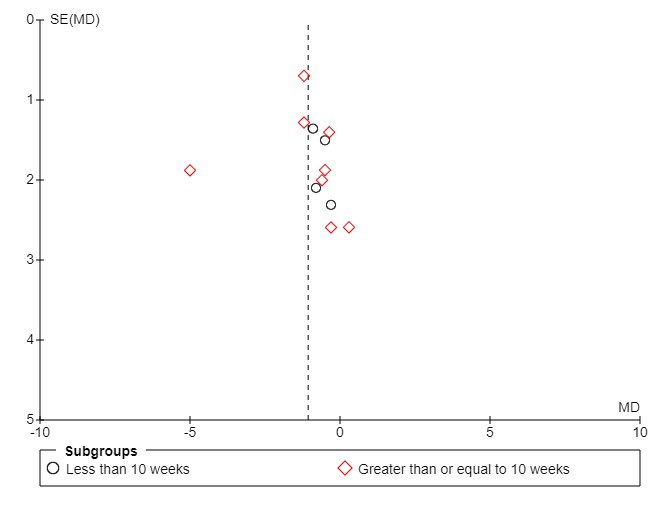


Figure S3B - Resistance Training vs. Aerobic Training for Fat Mass


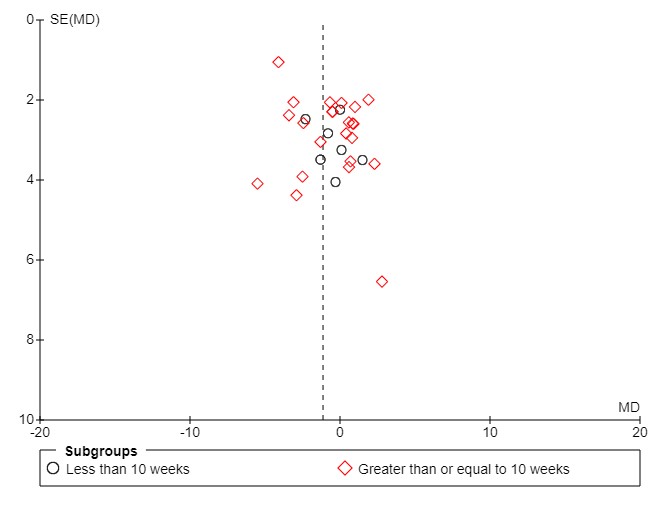


Figure S4B - Aerobic Training vs. Concurrent Training for Weight Loss


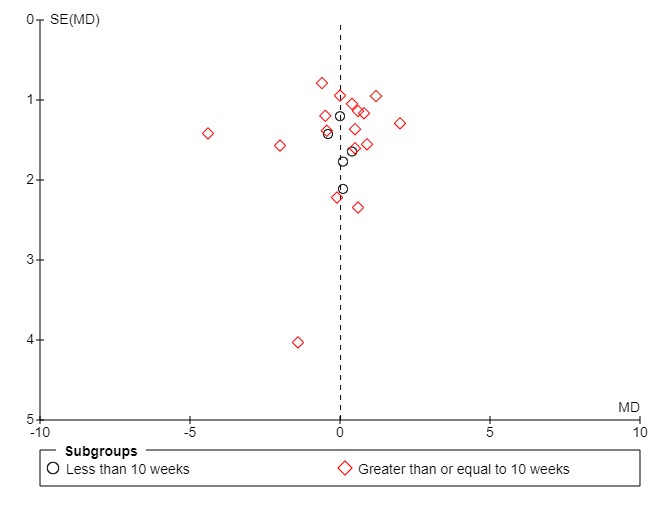


Figure S5B - Aerobic Training vs. Concurrent Training for % Body Fat


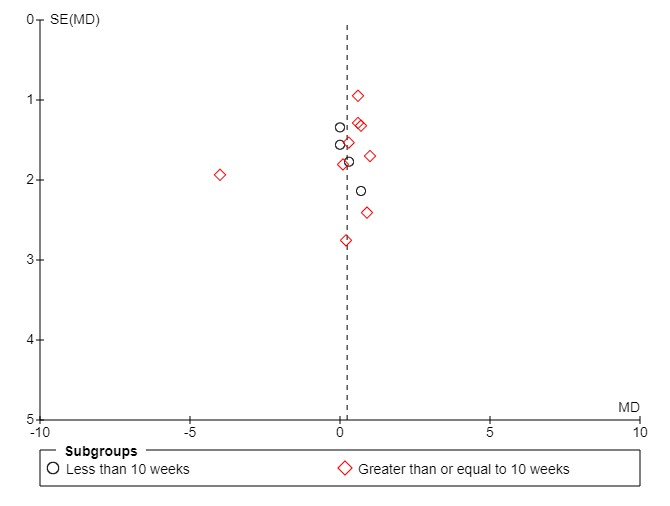


Figure S6B - Aerobic Training vs. Concurrent Training Fat Mass


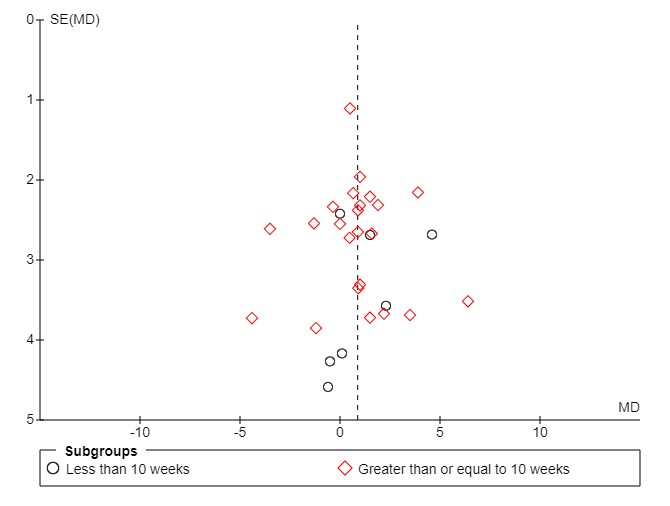


Figure S7B - Concurrent Training vs. Resistance Training for Weight Loss


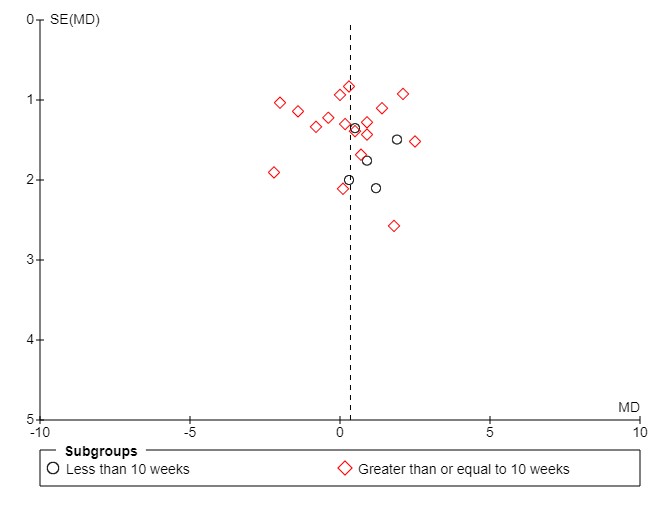


Figure S8B - Concurrent Training vs. Resistance Training for % Body Fat


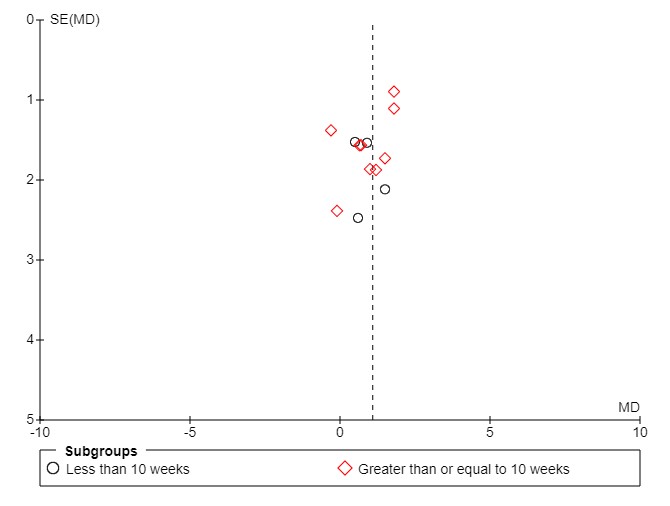


Figure S9B - Concurrent Training vs. Resistance Training for Fat Mass
