# Supplementary material for: Comparison of concurrent, resistance, or aerobic training on body fat loss: a systematic review and meta-analysis
Source: J Int Soc Sports Nutr. 2025 May 22;22(1):2507949. doi: 10.1080/15502783.2025.2507949 (PMC12107660; doi:10.1080/15502783.2025.2507949)

Resistance Training (RT) vs. Aerobic Training (AT): Body Mass Loss


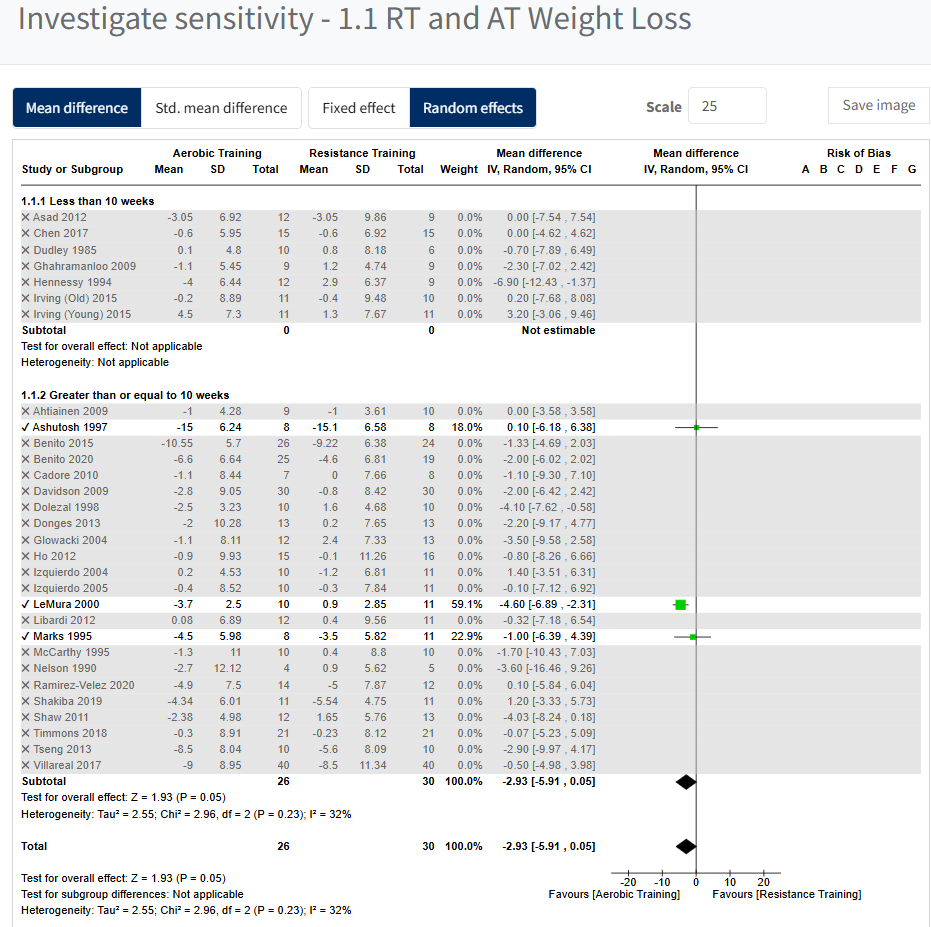


RT vs. AT: Body Fat Percentage Loss


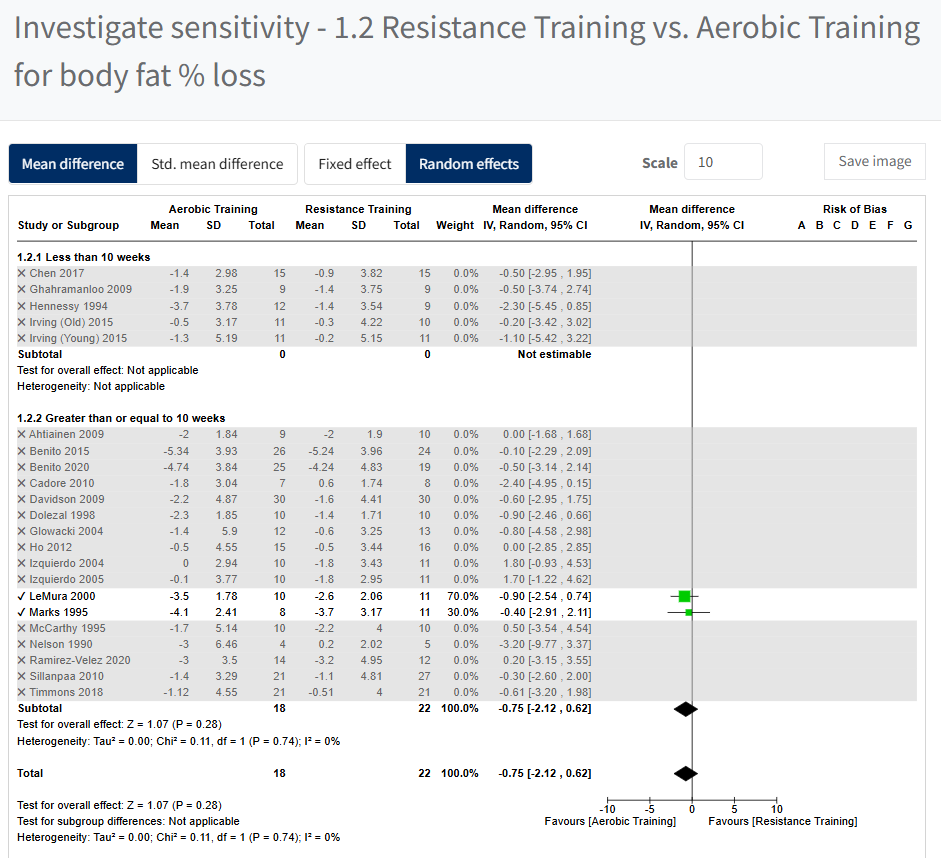


RT vs. AT: Fat Mass Loss


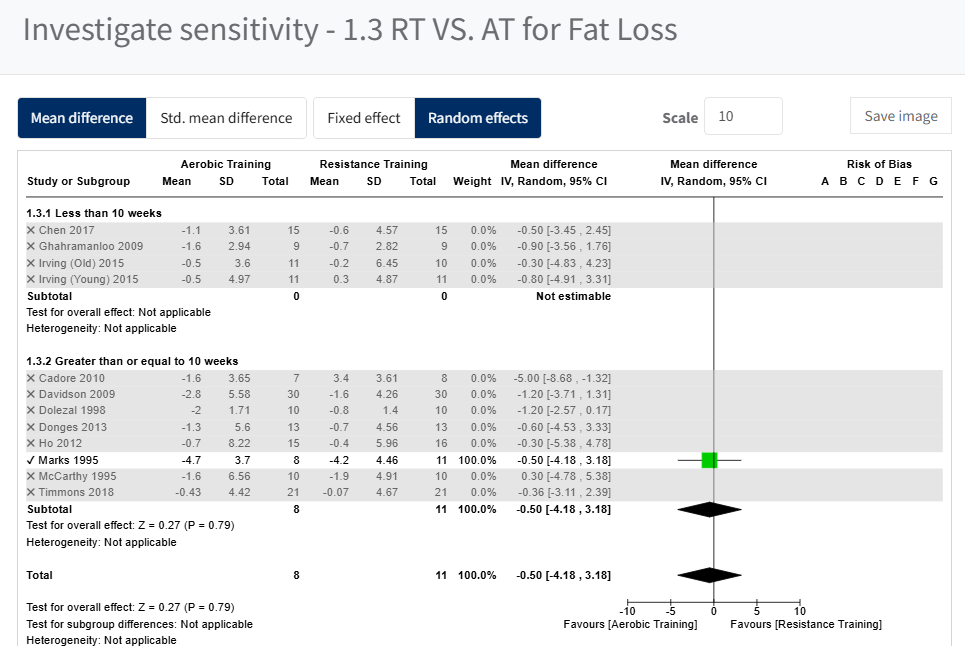


RT vs. AT: Fat-Free Mass Changes


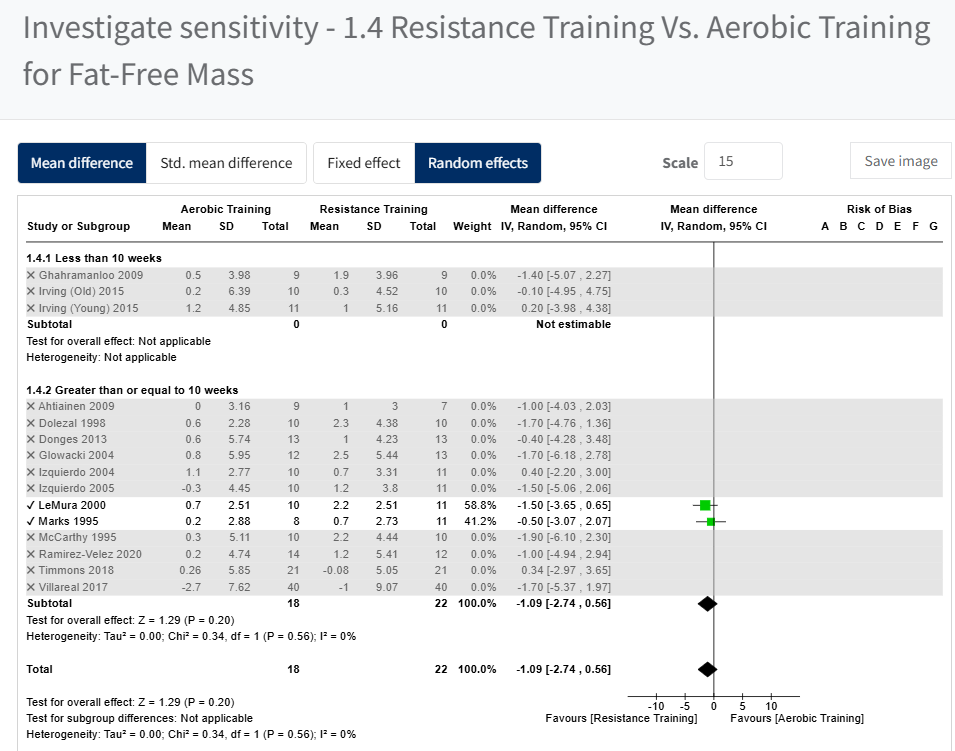


AT vs. Concurrent Training (CT): Body Mass Loss


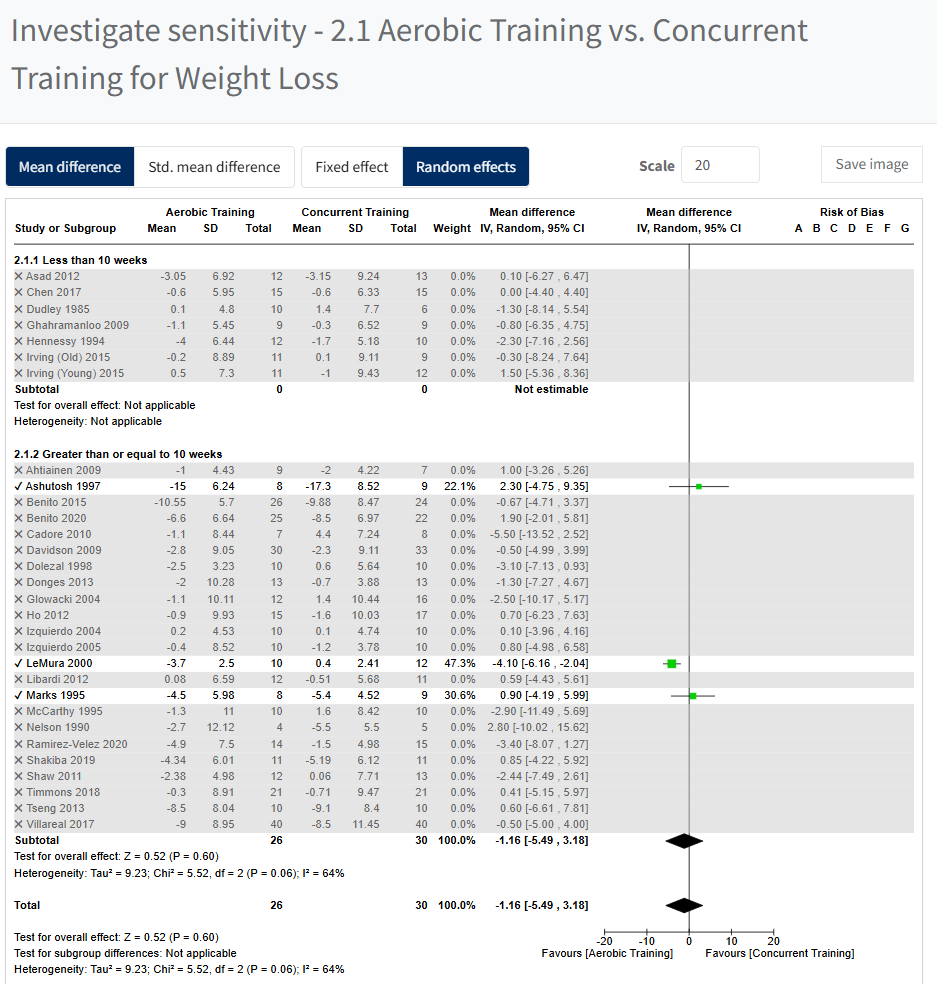


AT vs. CT: Body Fat Percentage Loss


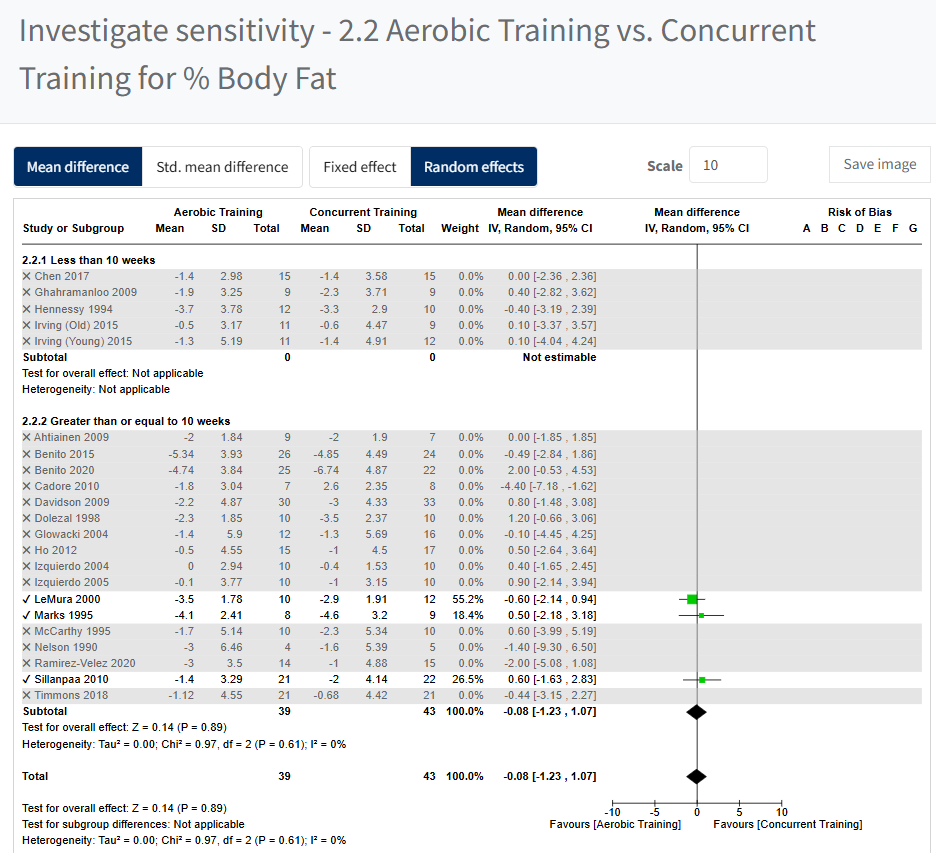


AT vs. CT: Fat Mass Loss


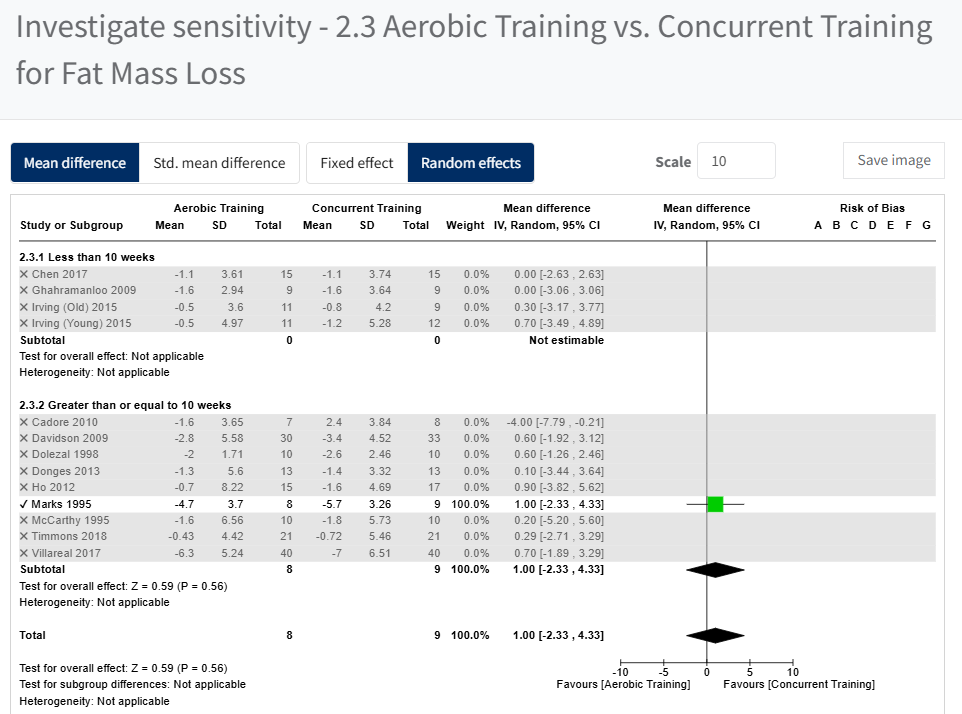


AT vs. CT: Fat-Free Mass Changes


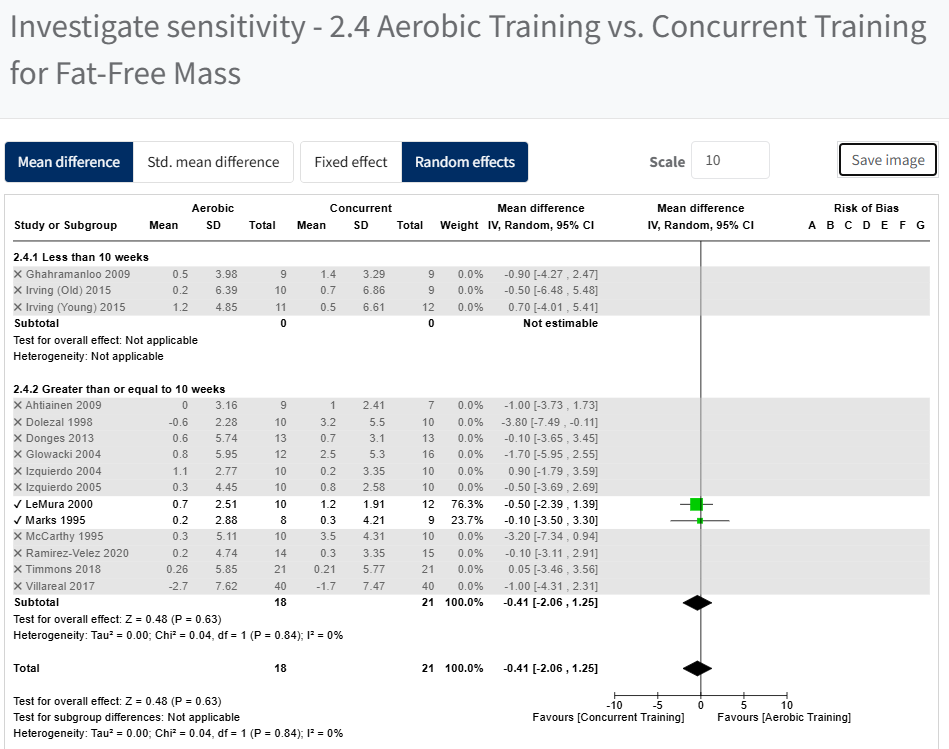

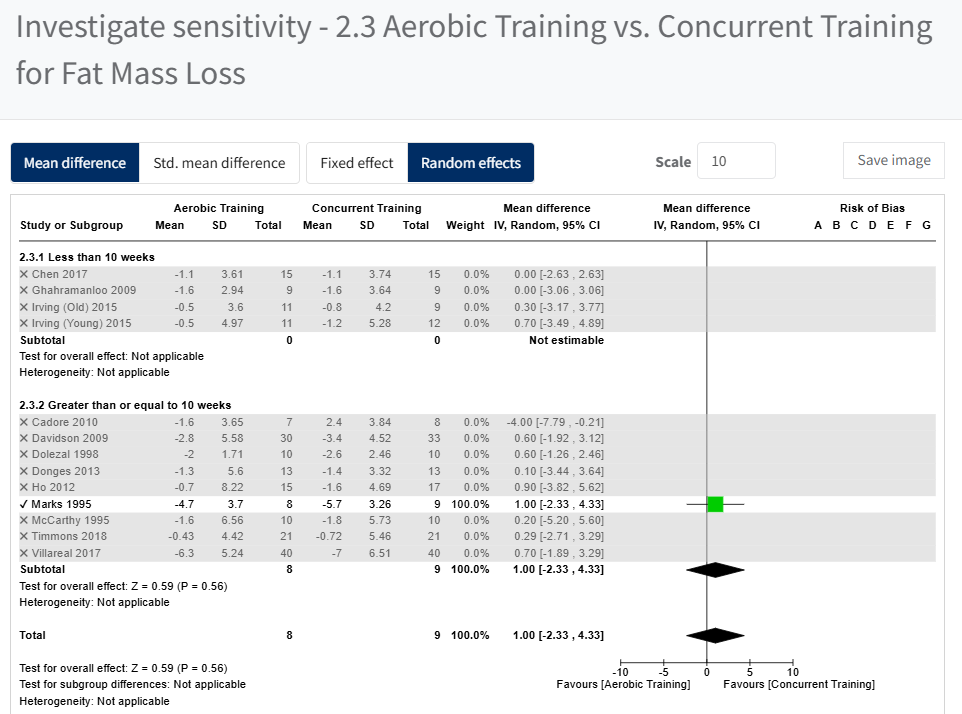


CT vs. RT: Body Mass Loss


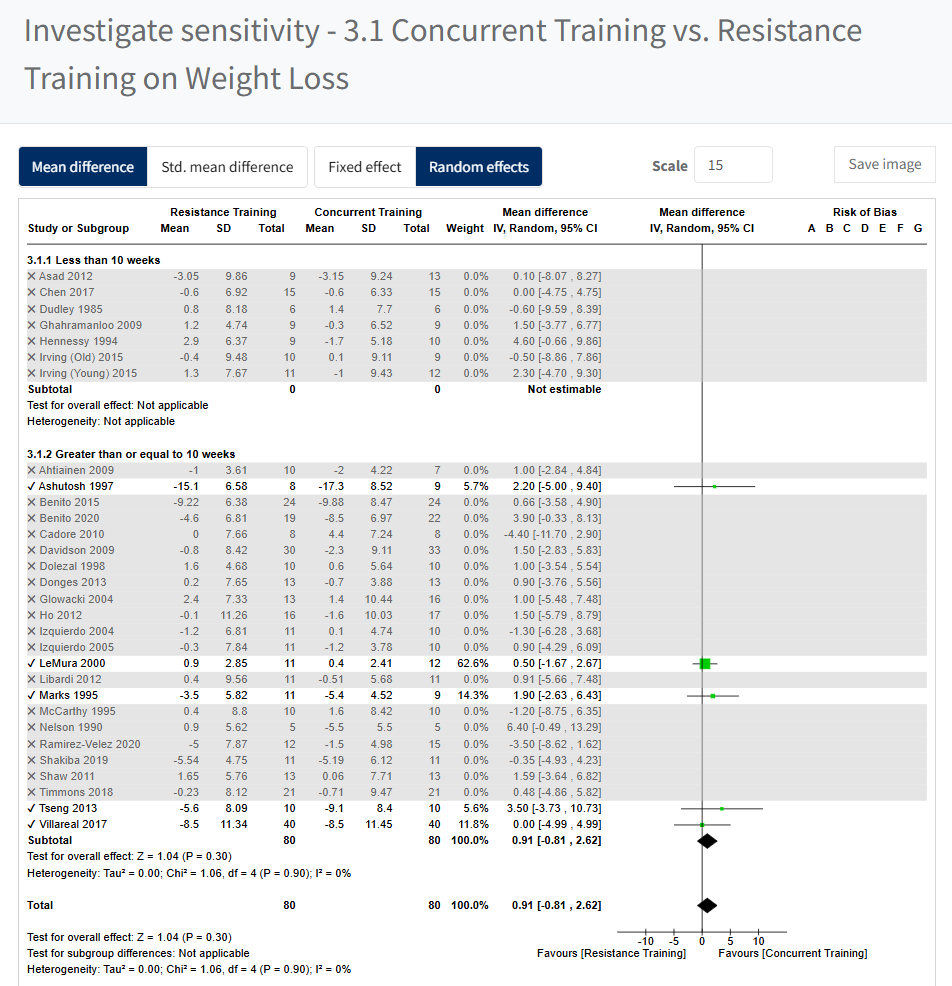


CT vs. RT: Body Fat Percentage Loss


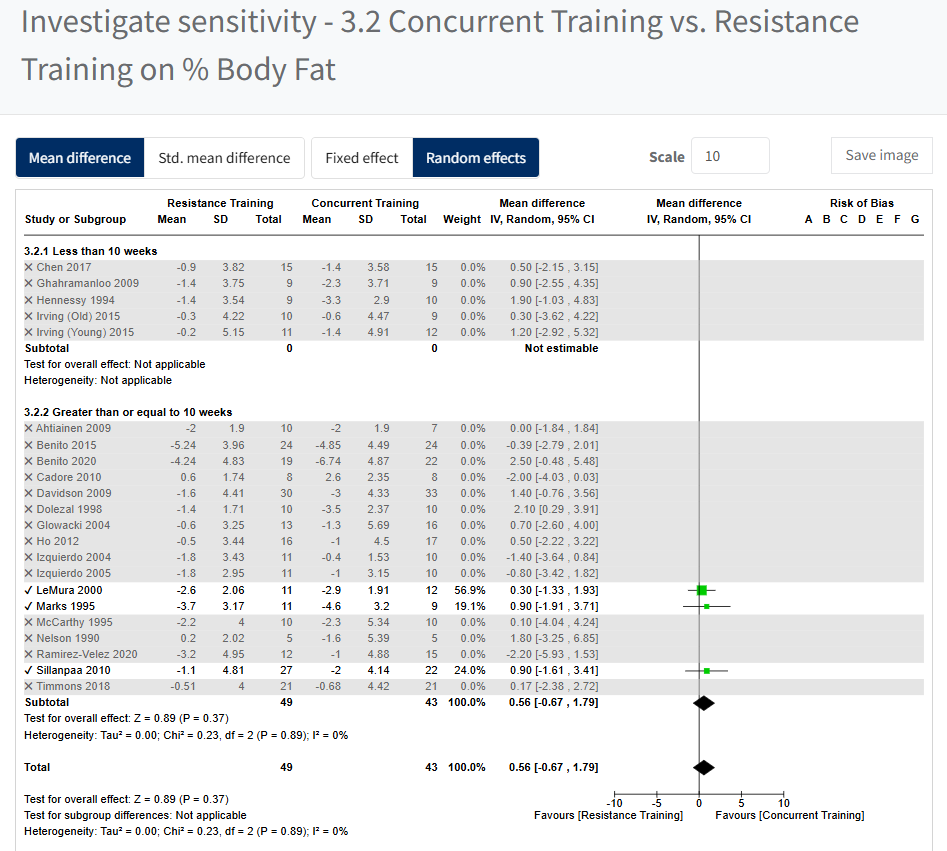


CT vs. RT: Fat Mass Loss


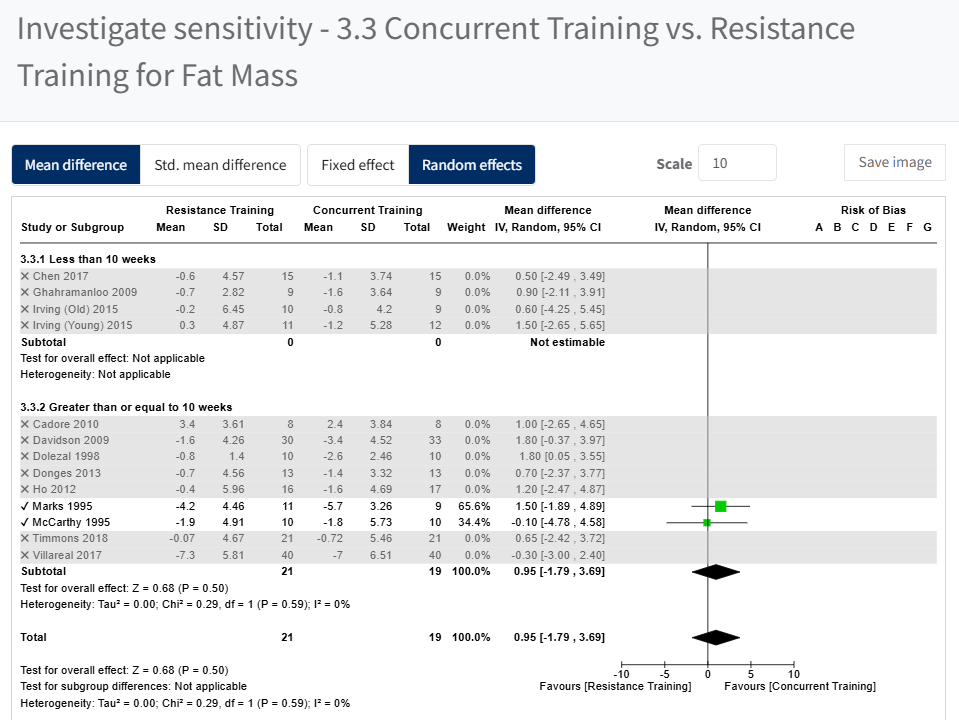


CT vs. RT: Fat-Free Mass Changes


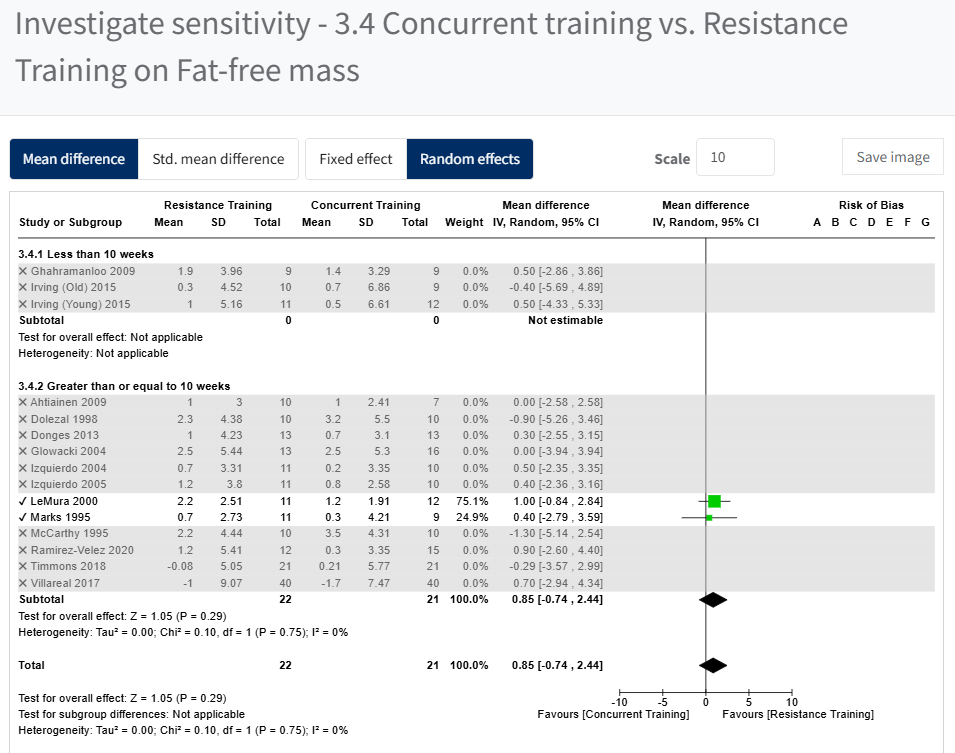

Supplement: Supplemental Material [file RSSN_A_2507949_SM0656.zip › Supp/CRAB Study Appendix C_RevisedSubmission.docx]
