# Supplementary material for: Comparison of concurrent, resistance, or aerobic training on body fat loss: a systematic review and meta-analysis
Source: J Int Soc Sports Nutr. 2025 May 22;22(1):2507949. doi: 10.1080/15502783.2025.2507949 (PMC12107660; doi:10.1080/15502783.2025.2507949)
Supplement: Supplemental Material [file RSSN_A_2507949_SM0656.zip › Supp/CRAB_Study_Appendix_A_RevisedSubmissionclean.docx]

| **Table S1A. PubMed search string concept chart.** | |
| --- | --- |
| **Concept #1: Concurrent Training** | **Concept #2: Fat Loss (not MH)** |
| “Resistance Trained” [NL] 1,332 | Adipose Tissue [MH] 108,376 |
| ("resistance"[All Fields] AND "training"[All Fields]) 30,382 | “Adipose Tissue” [NL] 137,373 |
| ("strength"[All Fields] AND "training"[All Fields]) 32,557 | Body Composition [MH] 62,066 |
| “Strength Trained” [NL/All Fields] 442 | “Body Composition” [NL] 66,903 |
| ("strength"[All Fields] AND "program"[All Fields]) 24,189 | ("body"[All Fields] AND "fat"[All Fields]) 122,794 |
| “Strength Programs” [All Fields/NL] 22 | Body Fat Distribution [MH] 17,299 |
| “Strength Programmes” [All Fields/NL] 8 | “Body Fat Distribution” [NL] 5,167 |
| "strength program*"[All Fields] 84 | “Body fat Percentage” [NL/All Fields] 4,635 |
| “strengthening program*” 539 | “Body mass index” [All Fields] 282,598 |
| ("weight"[All Fields] AND "lifting"[All Fields]) 6,959 | Body weight [MH] 514,087 |
| “weightlifting” [All Fields] 1,078 | “Body weight” [NL] 372,992 |
| ("weightlifting"[All Fields]) AND ("program*") 197 | “Fatty Tissue” [NL or All Fields] 3,349 |
| (("weight") AND ("lifting")) AND ("program*") 1,235 | “Percent Body Fat” [All Fields] 3,709 |
| ((("weight") AND ("lifting")) AND ("exercise")) AND ("program*") 833 | “Percent of Body Fat” [NL/All Fields] 140 |
|  | “Percentage of Body Fat” [NL/All Fields] 1,934 |
| ("endurance"[All Fields] AND "training"[All Fields]) 17,901 | (("percent body fat"[All Fields]) OR ("percent of body fat"[All Fields])) OR ("percentage of body fat"[All Fields]) 5,707 |
| “Endurance Trained” [NL or All Fields] 1,974 | “Subcutaneous Fat” [NL or All Fields] 14,340 |
| “aerobic train*” 3043 | “Total Body Fat” [NL] 3,272 |
| “Aerobically Trained” [NL or All Fields] 216 |  |
| ("aerobic"[All Fields] AND "exercise"[All Fields]) 28,458 | Weight Loss [MH] 47,269 |
| Physical Endurance [MH] 36,614 | “Weight Loss” [NL] 113,830 |
| ("physical"[All Fields] AND "endurance"[All Fields]) 31,792 | “Weight Reduction” [NL] 12,971 |
| ("physical"[All Fields] AND "exertion"[All Fields]) 62,588 | “Weight Reduction Program” [NL] 435 |
| Physical Fitness [MH] 35,109 | “Weight Reduction Programme” [All Fields] 104 |
| “Physical Fitness” [NL or All Fields] 35,466 | Weight Reduction Programs [MH] 2,975 |
| ("physical"[All Fields] AND "fitness"[All Fields]) 50,985 | “Weight Reduction Programs” [NL] 2,905 |
| ("physical"[All Fields] AND "education"[All Fields] AND "training"[All Fields]) 38,799 | “Weight Reduction Programmes” [NL] 57 |
|  | “weight reduction program*” 3,420 |
| "Combined resistance and aerobic exercise"[All Fields] 35 |  |
| “Combined strength endurance” [All Fields/NL] 7 |  |
| “Combined resistance endurance” [All Fields/NL] 9 |  |
| “Combined resistance exercise” [NL/All Fields] 12 |  |
| “Combined resistance endurance training” [All Fields/NL] 5 |  |
| “Concurrent Exercise” [All Fields] 165 |  |
| “Concurrent Endurance Exercise” [All Fields] 3 |  |
| “Concurrent Endurance Training” [All Fields] 5 |  |
| “Concurrent Resistance Training” [All Fields] 7 |  |
| “Concurrent Strength Training” [All Fields] 5 |  |
| "endurance"[All Fields] AND "strength training"[All Fields] |  |
| “Endurance and Resistance Training” [All Fields] 144 |  |
| “Interference Effect” [All Fields] 1,944 |  |
| "simultaneous training"[All Fields] 70 |  |
| ((strength OR endurance) AND ("simultaneous training"[All Fields])) 10 |  |
| “Strength and Endurance Training” [All Fields] 239 |  |
| "concurrent*"[All Fields] AND ("strength"[All Fields] OR "strengths"[All Fields]) AND ("endurance training"[MeSH Terms] OR ("endurance"[All Fields] AND "training"[All Fields]) OR "endurance training"[All Fields]) 299 | **Conceptual Filter (Conceptual Concept #3)** |
| ("strength"[All Fields] OR "strengths"[All Fields]) AND ("endurance training"[MeSH Terms] OR ("endurance"[All Fields] AND "training"[All Fields]) OR "endurance training"[All Fields]) 5,624 | ("randomized controlled trial*"[All Fields] OR "randomised controlled trial*"[All Fields] OR "rct"[All Fields]) OR ("Randomized Controlled Trial" [Publication Type] OR "Randomized Controlled Trials as Topic"[Mesh]) |

PubMed Final Search with Limits

Resistance Training OR Endurance Training OR Concurrent Training AND Body Fat AND Randomized Controlled Trials

With these 4 PubMed Filters:

- Years 1980 to Current
- Humans
- English Language
- Adult 19+ years

((((((((((((((((("resistance trained"[All Fields]) OR (("resistance"[All Fields] AND "training"[All Fields]))) OR (("strength"[All Fields] AND "training"[All Fields]))) OR ("strength trained"[All Fields])) OR (("strength"[All Fields] AND "program"[All Fields]))) OR ("strength programs"[All Fields])) OR ("strength programmes"[All Fields])) OR ("strength program*")) OR ("strengthening program*")) OR (("weight"[All Fields] AND "lifting"[All Fields]))) OR ("weightlifting"[All Fields])) OR (("weightlifting"[All Fields]) AND ("program*"))) OR ((("weight") AND ("lifting")) AND ("program*"))) OR (((("weight") AND ("lifting")) AND ("exercise")) AND ("program*"))) OR (((((((((((((("endurance"[All Fields] AND "training"[All Fields])) OR ("endurance trained"[All Fields])) OR ("aerobic train*")) OR ("aerobically trained"[All Fields])) OR (("aerobic"[All Fields] AND "exercise"[All Fields]))) OR ("physical endurance"[MeSH Terms])) ) OR (("physical"[All Fields] AND "endurance"[All Fields]))) OR (("physical"[All Fields] AND "exertion"[All Fields]))) OR ("physical fitness"[MeSH Terms])) OR ("physical fitness"[All Fields])) OR (("physical"[All Fields] AND "fitness"[All Fields]))) OR (("physical"[All Fields] AND "education"[All Fields] AND "training"[All Fields])))) OR ((((((((((((((((("combined resistance and aerobic exercise"[All Fields]) OR ("combined strength endurance"[All Fields]) OR ("combined resistance endurance"[All Fields])) OR ("combined resistance exercise"[All Fields])) OR ("combined resistance endurance training"[All Fields])) OR ("concurrent exercise"[All Fields])) OR ("concurrent endurance exercise"[All Fields])) OR ("concurrent endurance training"[All Fields])) OR ("concurrent resistance training"[All Fields])) OR ("concurrent strength training"[All Fields])) OR ("endurance"[All Fields] AND "strength training"[All Fields])) OR ("endurance and resistance training"[All Fields])) OR ("interference effect"[All Fields])) OR ("simultaneous training"[All Fields])) OR (((strength OR endurance) AND ("simultaneous training"[All Fields])))) OR ("strength and endurance training"[All Fields])) OR ("concurrent*"[All Fields] AND ("strength"[All Fields] OR "strengths"[All Fields]) AND ("endurance training"[MeSH Terms] OR ("endurance"[All Fields] AND "training"[All Fields]) OR "endurance training"[All Fields]))) OR (("strength"[All Fields] OR "strengths"[All Fields]) AND ("endurance training"[MeSH Terms] OR ("endurance"[All Fields] AND "training"[All Fields]) OR "endurance training"[All Fields])))) AND ((((((((((((((((((((((((((("adipose tissue"[MeSH Terms]) OR ("adipose tissue"[All Fields])) OR ("body composition"[MeSH Terms])) OR ("body composition"[All Fields])) OR (("body"[All Fields] AND "fat"[All Fields]))) OR ("body fat distribution"[MeSH Terms])) OR ("body fat distribution"[All Fields])) OR ("body fat percentage"[All Fields])) OR ("body mass index"[All Fields])) OR ("body weight"[MeSH Terms])) OR ("body weight"[All Fields])) OR ("fatty tissue"[All Fields])) OR ("percent body fat"[All Fields])) OR ("percent of body fat"[All Fields])) OR ("percentage of body fat"[All Fields])) OR ((("percent body fat"[All Fields]) OR ("percent of body fat"[All Fields])) OR ("percentage of body fat"[All Fields]))) OR ("subcutaneous fat"[All Fields])) OR ("total body fat"[All Fields])) OR ("weight loss"[MeSH Terms])) OR ("weight loss"[All Fields])) OR ("weight reduction"[All Fields])) OR ("weight reduction program"[All Fields])) OR ("weight reduction programme"[All Fields])) OR ("weight reduction programs"[MeSH Terms])) OR ("weight reduction programs"[All Fields])) OR ("weight reduction programmes"[All Fields])) OR ("weight reduction program*"))) AND (("randomized controlled trial*"[All Fields] OR "randomised controlled trial*"[All Fields] OR "rct"[All Fields]) OR ("Randomized Controlled Trial" [Publication Type] OR "Randomized Controlled Trials as Topic"[Mesh]))

**Note:** We did not include “randomized controlled trial” as a PubMed filter since this was used as a ‘conceptual filter’ (i.e., a search term) in the search string)
